# SALL1 enforces microglia-specific DNA binding and function of SMADs to establish microglia identity

**DOI:** 10.1038/s41590-023-01528-8

**Published:** 2023-06-15

**Authors:** Bethany R. Fixsen, Claudia Z. Han, Yi Zhou, Nathanael J. Spann, Payam Saisan, Zeyang Shen, Christopher Balak, Mashito Sakai, Isidoro Cobo, Inge R. Holtman, Anna S. Warden, Gabriela Ramirez, Jana G. Collier, Martina P. Pasillas, Miao Yu, Rong Hu, Bin Li, Sarah Belhocine, David Gosselin, Nicole G. Coufal, Bing Ren, Christopher K. Glass

**Affiliations:** 1 Department of Cellular and Molecular Medicine, School of Medicine, UC San Diego, La Jolla, CA USA; 2 Department of Biochemistry and Molecular Biology, Nippon Medical School, Tokyo, Japan; 3 Department of Biomedical Sciences of Cells and Systems, Section Molecular Neurobiology, University of Groningen and University Medical Center Groningen, Groningen, the Netherlands; 4 Sanford Consortium for Regenerative Medicine, La Jolla, CA USA; 5 Ludwig Institute for Cancer Research, La Jolla, CA USA; 6 Axe Neuroscience, Centre de Recherche du CHU de Québec, Université Laval, Quebec, Quebec Canada; 7 Département de Médecine Moléculaire de la Faculté de Médecine, Université Laval, Quebec, Quebec Canada; 8 Department of Pediatrics, School of Medicine, UC San Diego, La Jolla, CA USA; 9 Department of Medicine, School of Medicine, UC San Diego, La Jolla, CA USA

**Keywords:** Gene regulation in immune cells, Neuroimmunology

## Abstract

Spalt-like transcription factor 1 (SALL1) is a critical regulator of organogenesis and microglia identity. Here we demonstrate that disruption of a conserved microglia-specific super-enhancer interacting with the *Sall1* promoter results in complete and specific loss of *Sall1* expression in microglia. By determining the genomic binding sites of SALL1 and leveraging *Sall1* enhancer knockout mice, we provide evidence for functional interactions between SALL1 and SMAD4 required for microglia-specific gene expression. SMAD4 binds directly to the *Sall1* super-enhancer and is required for *Sall1* expression, consistent with an evolutionarily conserved requirement of the TGFβ and SMAD homologs *Dpp* and *Mad* for cell-specific expression of *Spalt* in the *Drosophila* wing. Unexpectedly, SALL1 in turn promotes binding and function of SMAD4 at microglia-specific enhancers while simultaneously suppressing binding of SMAD4 to enhancers of genes that become inappropriately activated in enhancer knockout microglia, thereby enforcing microglia-specific functions of the TGFβ–SMAD signaling axis.

## Abstract (web summary)

Glass and colleagues show that the transcription factor SALL1-associated super-enhancer is exclusively activated in microglia, in part through SMAD4-mediated signaling, and that SALL1 subsequently enforces microglia-specific functions of SMAD4.

## Main

Microglia, the major tissue-resident macrophage (TRM) population of the central nervous system, are self-renewing, yolk sac-derived cells whose functions include regulation of brain development, maintenance of neural circuitry, and response to injury/infection^1^. Like other TRMs, microglia assume a spectrum of activation states and phenotypes in response to environmental signals and perturbations. In addition to their adaptive functions, numerous studies have implicated microglia as playing pathogenic roles in neurodevelopmental, psychiatric and neurodegenerative diseases^2^. Unlike many populations of TRMs outside of the brain, microglia are not replaced by bone-marrow-derived macrophage precursors following birth under normal conditions.

Spalt-like transcription factor 1 (SALL1), a zinc-finger transcription factor (TF), was recently identified through a loss-of-function study as a key transcriptional regulator of microglia identity and phenotype in the mouse^3^. Members of the Spalt family of TFs are highly conserved in metazoan organisms and play diverse roles in organ development. Heterozygous loss-of-function mutations of SALL1 in humans lead to Townes–Brock syndrome^4,5^, while *Sall1* deletion in mice results in perinatal lethality due to severe kidney defects^6^. In the mouse, *Sall1* expression is induced between embryonic days 11 and 12 in yolk sac-derived hematopoietic progenitor cells (HPCs) that have entered the developing brain and are destined to become resident microglia^7,8^. Expression of *Sall1* is dependent on TGFβ1 signaling, which is broadly required for microglia differentiation and survival^8,9^. *Sall1* expression, in concert with many other microglia-specific genes, falls rapidly and dramatically when microglia are transferred from the brain to an in vitro environment, indicating a continuous requirement for brain environmental signals to maintain an in vivo microglia phenotype^10–12^.

In this Resource, we show that *Sall1* expression in microglia is regulated by a microglia-specific super-enhancer (SE), and that disruption of this gene regulatory element results in a selective loss of *Sall1* expression in microglia. We define the genome-wide binding of SALL1 and leverage the enhancer knockout (EKO) model to examine the transcriptional effects of SALL1, revealing that SALL1 is functioning as both an activator and a repressor in microglia. We provide evidence that signaling through SMAD4 maintains expression of *Sall1*, which in turn enforces a microglia-specific DNA binding program of SMAD4 at key gene regulatory elements associated with microglia identity and function.

## Results

### Microglia *Sall1* expression is regulated by an SE

**Fig. 1:**
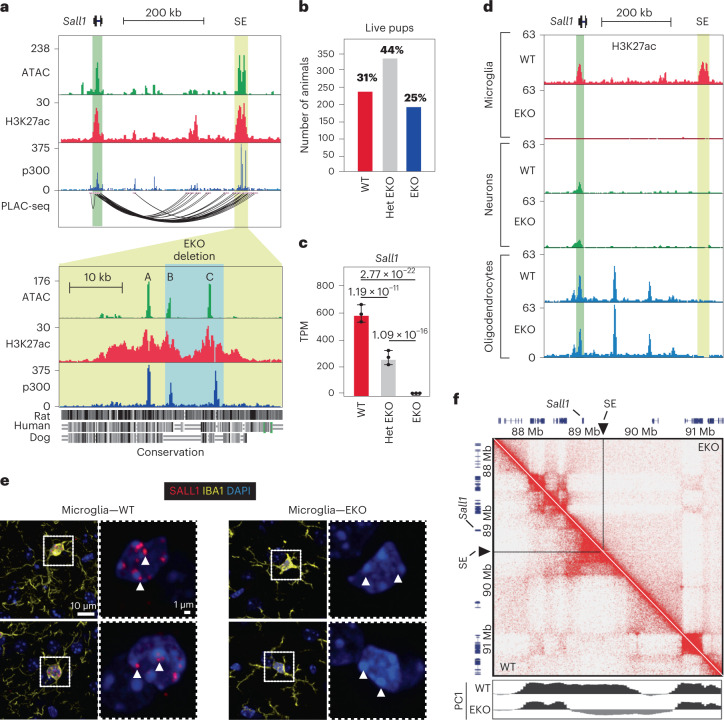
*Sall1* expression is regulated by a microglia-specific SE. **a**, Genome browser tracks of ATAC-seq (sorted live microglia), H3K27ac ChIP and p300 ChIP (sorted PU1 + nuclei), in addition to PLAC-seq signal at the *Sall1* locus. Green shading, *Sall1* gene. Yellow shading, *Sall1* SE. Labels A, B and C denote the three main regions of open chromatin in the SE. Blue shading, region encompassing the *Sall1* SE KO. *n* ≥ 2 per assay. See also Extended Data Fig. 1. **b**, Counts of WT, Het EKO and EKO pups after weaning. **c**, Bar plots for *Sall1* expression in WT, Het EKO and EKO microglia (*n* = 3 mice/genotype). Data are represented as mean with standard deviation; p-adj from DESeq2 analysis (Wald’s test with multiple testing correction using Benjamini–Hochberg method). **d**, Genome browser tracks of H3K27ac ChIP in EKO and WT brain nuclei at the *Sall1* locus. Microglia, sorted PU1^+^ nuclei; neurons, sorted NeuN^+^ nuclei; oligodendrocytes, sorted Olig2^+^ nuclei. Green shading, *Sall1* gene. Yellow shading, *Sall1* SE. Tracks represent combined normalized tag counts; *n* ≥ 2 per genotype/cell type. **e**, Representative confocal images of frontal cortical regions of WT and EKO brains from 6-week-old mice (*n* = 3 per genotype) showing DAPI, IBA1 and SALL1. White arrowheads denote location of SALL1 puncta in WT and lack of puncta in EKO. Between 120 and 150 microglia were assessed morphologically for each sample. See also Extended Data Figs. 2 and 3. **f**, Hi-C contact frequency map at the *Sall1* locus in WT and EKO microglia, normalized by coverage (*n* = 2 per genotype). PC1 values denote ‘A’ euchromatin compartment (black) and ‘B’ heterochromatin compartment (gray).

**Extended Data Fig. 1:**
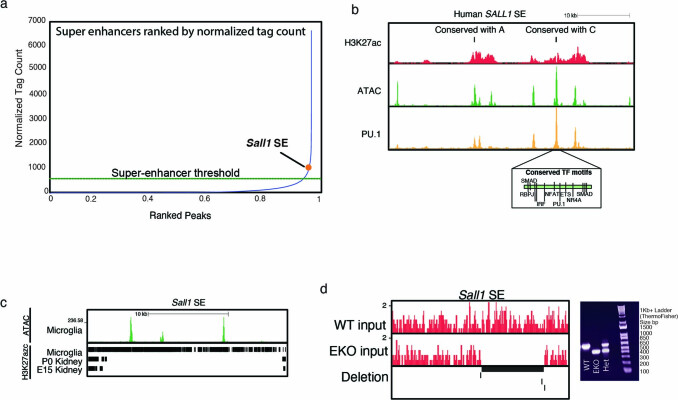
Deletion of the *Sall1* super-enhancer. **a**. Plot of WT microglia enhancers ranked by normalized H3K27ac tag count. Dotted line represents the cutoff for an enhancer to be considered a super-enhancer. **b**. Genome browser of the human *SALL1* super-enhancer with H3K27ac ChIP, ATAC, and PU.1 ChIP. Regions conserved with the mouse *Sall1* super-enhancer Region A and Region C are marked above the H3K27ac. Conserved TF binding sites are annotated in the region homologous to mouse Region C. **c**. Genome browser of the mouse *Sall1* super-enhancer and the overlap of mouse H3K27ac in microglia and embryonic/early postnatal kidney. **d**. Genome browser showing input DNA from microglia and the *Sall1* SE deletion (n = 4–6 mice/geno- type over > 3 experiments). Primers used for genotyping are marked below, and results from geno- typing are shown on the right. Genotyping was performed for all mice utilized in this study (>40 mice over >12 experiments).

To identify regions of open and active chromatin that may be putative enhancers regulating *Sall1* transcription in microglia, we performed assay for transposase-accessible chromatin with sequencing (ATAC-seq), chromatin immunoprecipitation followed by sequencing (ChIP–seq) for histone H3 lysine 27 acetylation (H3K27ac), a histone modification associated with active enhancers and promoters^12^, and ChIP–seq for p300, a transcriptional co-activator (Fig. 1a). ATAC-seq was performed in sorted microglia defined as CD11b^+^CD45^low^CX3CR1^+^ as previously described^10^. ChIP–seq for H3K27ac was performed using sorted PU.1^+^ nuclei^13^. We located a region located approximately −300 kb from the *Sall1* promoter that was marked by a cluster of high levels of open chromatin, H3K27ac and p300, which meets criteria described for SEs, a class of regulatory elements known to control cell identity-defining genes (Fig. 1a, yellow highlight; Extended Data Fig. 1a)^14–16^. We performed proximity ligation-assisted ChIP–seq (PLAC-seq) using histone H3 lysine 4 trimethyl (H3K4me3) to detect interactions between active promoters and putative enhancers^17,18^, thereby allowing identification of target genes of enhancers and SEs. The SE proximal to *Sall1* loops solely to the *Sall1* gene (Fig. 1a), similar to what is observed for the human microglia *SALL1* gene and its putative enhancer region^18^. Regions A and C of the *Sall1* SE contain sequences with ~75% homology to open chromatin regions in the human microglia *SALL1* SE (Extended Data Fig. 1b). Region C from mouse microglia overlaps the most prominent region of open chromatin and the most robust binding site of the microglia lineage determining transcription factor (LDTF) PU.1 in the human *SALL1* SE (Extended Data Fig. 1b). This site also contains conserved TF binding motifs for SMADs, NR4A, PU.1, ETS, IRF and RBPJ (Extended Data Fig. 1b), suggesting that this region may be a point of convergence of multiple cellular signaling pathways that regulate *Sall1* expression. Since SALL1 is a critical regulator of kidney development, we examined H3K27ac datasets from mouse embryonic day 15 and early postnatal kidney and found no overlap between the microglia SE and kidney H3K27ac signal (Extended Data Fig. 1c).

We utilized CRISPR/Cas9-mediated deletion to generate mice with a homozygous knockout (KO) spanning 13 kb of the SE (EKO) (Fig. 1a, blue highlight). The deletion was confirmed by sequencing of microglia input DNA, and polymerase chain reaction (PCR) (Extended Data Fig. 1d). Unlike previously reported *Sall1* null mice, EKO mice survive after birth (Fig. 1b) and through adulthood. Using RNA sequencing (RNA-seq), we found that levels of *Sall1* transcript in microglia are affected in an enhancer dosage-dependent manner, with a 50% reduction of *Sall1* messenger RNA in heterozygous EKO mice (Het EKO) and a complete loss of *Sall1* mRNA in EKO mice (Fig. 1c). EKO led to complete loss of H3K27ac signal at the *Sall1* locus in microglia, while H3K27ac signal at *Sall1* in other brain cell types known to express *Sall1*, such as oligodendrocytes and neurons, was unaffected by the EKO (Fig. 1d).

**Extended Data Fig. 2:**
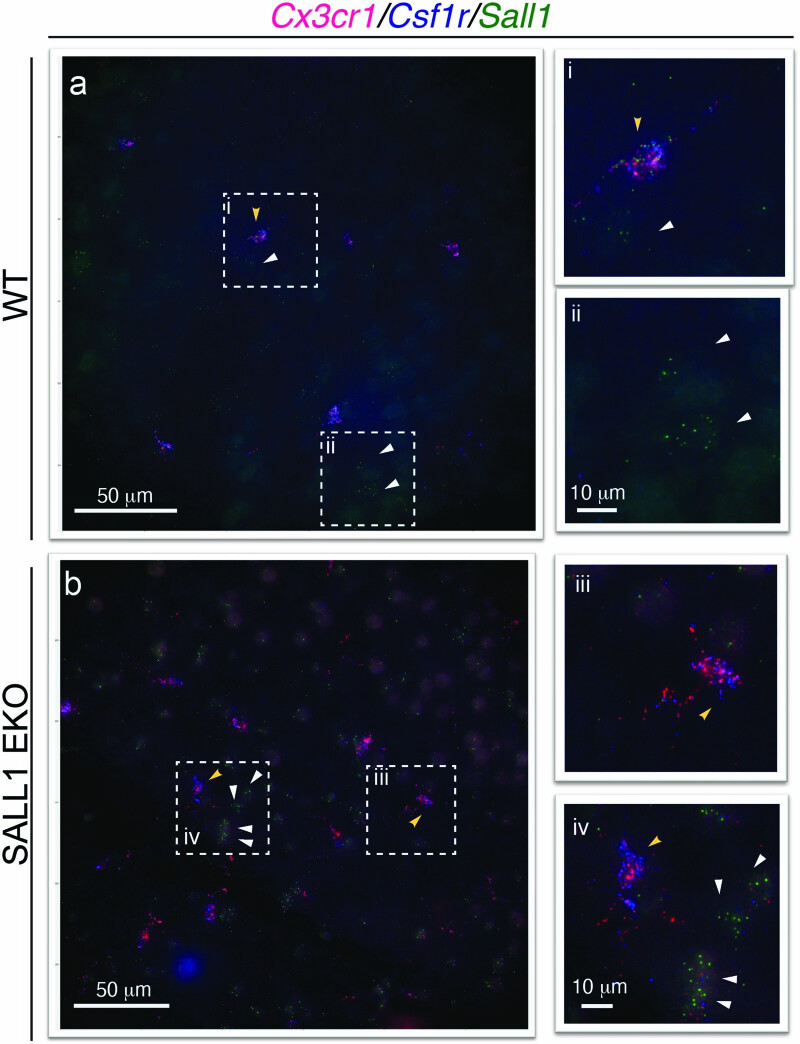
Single Molecule Fluorescence In Situ Hyrbidization (smFISH) for *Cx3cr1, Csf1r* and *Sall1* mRNA in WT and *Sall1* EKO brain sections. **a**. WT mice. Yellow arrowhead indicates *Sall1* mRNA expression in microglia as indicated by co-expression of *Cx3cr1* and *Csf1r* mRNA (inset i). White arrowhead indicates *Sall1* mRNA expression in cells lacking *Cx3cr1* and *Csf1r* mRNA (insets i and ii). **b**. SALL1 EKO mice. Yellow arrowheads indicate *Cx3cr1* and *Csfr1* expressing microglia that do not express *Sall1* mRNA (insets iii and iv). White arrowheads indicate cells that do not express *Cx3cr1* or *Csf1r* mRNA but do express *Sall1* mRNA (inset iv). for a,b - 225 ROI visualized per 4 total independent experiments.

**Extended Data Fig. 3:**
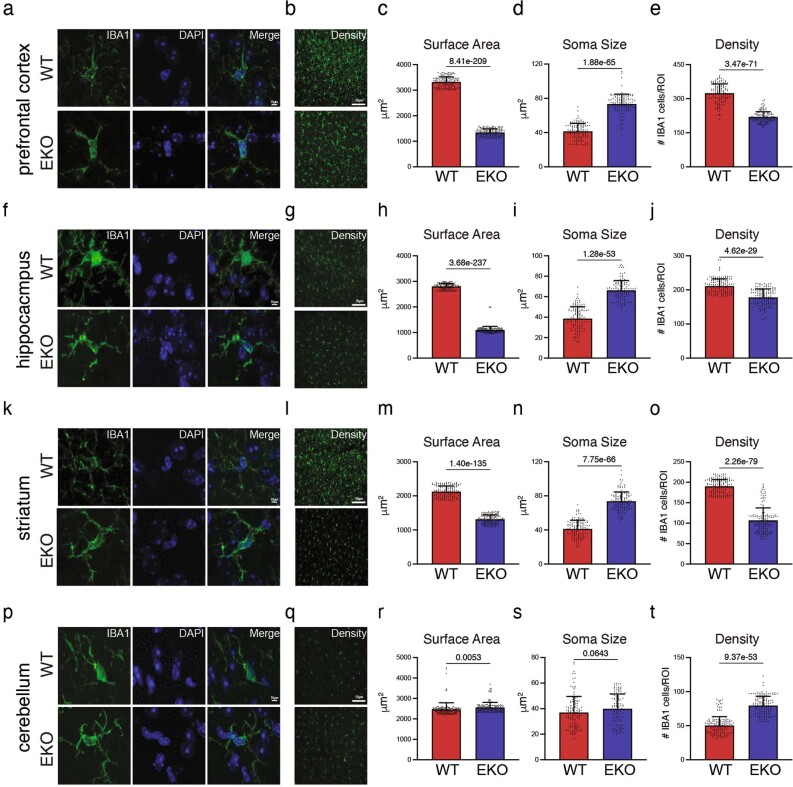
Quantitative analysis of microglia surface area, soma size and density in different brain regions of WT and SALL1 EKO mice. **a,b**. Representative brain section of the prefrontal cortex co-stained with IBA1 and DAPI. **c,d,e**. Prefrontal cortex - Quantification of surface area (n = 150 microglia/brain region/genotype), soma size (n = 124 microglia/brain region/geno- type), density (n = 142 microglia/brain region/genotype). **f,g**. Representative brain section of hippocampus co-stained with IBA1 and DAPI. **h,i,j**. Hippocampus - Quantification of surface area (n = 150 microglia/brain region/genotype), soma size (n = 124 microg- lia/brain region/genotype), density (n = 142 microglia/brain region/genotype). **k,l**. Representative brain section of striatum co-stained with IBA1 and DAPI. **m,n,o**. Striatum - Quantification of surface area (n = 150 microglia/brain region/genotype), soma size (n = 123 microg- lia/brain region/genotype), density (n = 150 microglia/brain region/genotype). **p,q**. Representative brain section of cerebellum co-stained with IBA1 and DAPI. **r,s,t**. Cerebellum - Quantification of surface area (n = 150 microglia/brain region/genotype), soma size (n = 123 microg- lia/brain region/genotype), density (n = 150 microglia/brain region/WT, n = 147 microglia/brain region/EKO). 15 ROIs, 4 sections per brain region, 2-3 mice/genotype. Unpaired two-tailed t-test with Welch’s correction was used to calculate significance. Data are represented as mean with s.d.

Immunofluorescence staining of SALL1 in whole mouse brain sections revealed that, in wild-type (WT) brain, IBA1-positive microglia robustly express SALL1 in the nucleus; multiple bright puncta corresponding to SALL1 localize to regions of heterochromatin, indicated by intense 4′,6-diamidino-2-phenylindole (DAPI) staining (Fig. 1e), consistent with what has been described in other cell systems^19,20^. A diffuse SALL1 staining pattern is also observed in the nucleus between heterochromatin regions. In contrast, brain sections of EKO mice do not exhibit either punctate or diffuse SALL1 staining in microglia nuclei (Fig. 1e), confirming antibody specificity. Single molecule fluorescence in situ hybridization documented lack of *Sall1* mRNA expression in EKO microglia, but maintenance of *Sall1* expression in other brain cell types, consistent with marks of active promoter and enhancer regions in neurons and oligodendrocytes (Extended Data Fig. 2). Microglia in EKO mice have notably decreased surface area, increased soma size and decreased density in the prefrontal cortex, hippocampus and striatum (Fig. 1e and Extended Data Fig. 3), consistent with prior studies of *Sall1* KO microglia^3,21^.

The complex staining pattern of SALL1 in microglia raised the question of whether it might play roles in genome organization, which has been proposed in past studies of SALL1 in other cell types^19,22^. To investigate consequences of the *Sall1* SE deletion on three-dimensional chromatin architecture, we performed in situ high-throughput chromatin conformation capture (Hi-C). In microglia isolated from WT mice, the *Sall1* locus was highly interconnected, forming a topological associated domain, consistent with the results of the PLAC-seq assay (Fig. 1a,f). In contrast, these interactions were almost completely lost in EKO microglia, with the corresponding PC1 values at the *Sall1* locus shifting from positive values associated with euchromatin-containing ‘A’ compartments (shaded black) to negative values associated with heterochromatin-containing ‘B’ compartments (shaded gray) (Fig. 1f). These results indicate that the 13 kb region deleted from the *Sall1* SE is essential for establishing the active regulatory features of this locus.

### Dose-dependent effects of reduced SALL1 gene expression

**Fig. 2:**
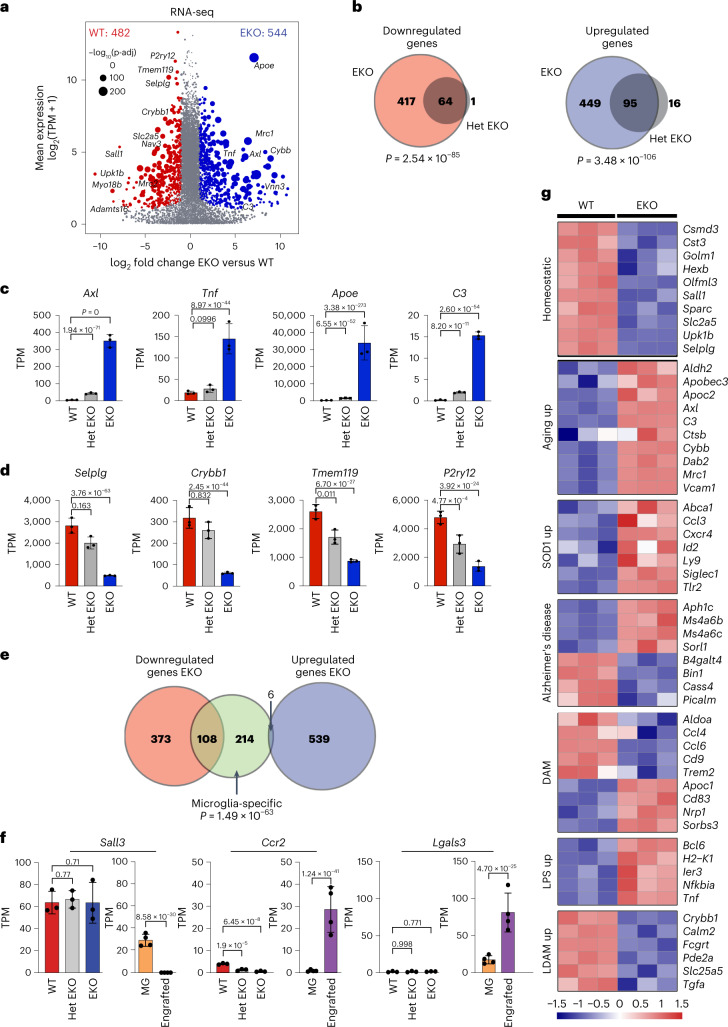
EKO microglia exhibit a loss of microglia identity and an increased signature of aging and inflammation. **a**, MA plot of RNA-seq data comparing WT and EKO microglia. *n* = 3 per group. DEGs (DESeq2 analysis with Wald’s test with multiple testing correction using Benjamini–Hochberg method) are defined as p-adj <0.05, FC >2 or <−2, and log_2_(TPM + 1) >2 in at least one group. **b**, Comparison of overlap between genes increased and decreased in EKO and Het EKO microglia as compared with WT microglia. *P* values were calculated using one-tailed Fisher exact test. See also Extended Data Fig. 4. **c**, Bar plots for expression of upregulated genes in WT as compared with Het EKO and EKO microglia. Red, WT; gray, Het EKO; blue, EKO. *n* = 3 per genotype. Data are represented as mean with standard deviation, p-adj from DESeq2 analysis (Wald’s test with multiple testing correction using Benjamini–Hochberg method) **d**, Bar plots for expression of downregulated genes in WT as compared with Het EKO and EKO microglia. Red, WT; gray, Het EKO; blue, EKO. *n* = 3 per genotype. Data are represented as mean with standard deviation, p-adj from DESeq2 analysis (Wald’s test with multiple testing correction using Benjamini–Hochberg method). **e**, Overlap of significantly downregulated and upregulated genes in EKO versus genes expressed more highly in microglia than other TRMs (Supplementary Table 1). *P* value for overlaps was calculated using one-tailed Fisher exact test. **f**, Bar plots for expression of DEGs between resident microglia (MG) and peripherally engrafted microglia-like cells from Shemer et al.^25^ (*n* = 4 per group), and in WT, Het EKO and EKO microglia from the present study (*n* = 3 per genotype). Data are represented as mean with standard deviation, p-adj from DESeq2 analysis (Wald’s test with multiple testing correction using Benjamini–Hochberg method). **g**, Heat map of DEGs (p-adj from DEseq2 <0.05) in EKO versus WT microglia that are associated with diverse microglia phenotypes (aging^29^, the SOD model of ALS^29^, AD risk genes^32^, DAM^30^, LPS-treated^29^, LDAMs^31^ and homeostatic microglia^10,11,39^). Each row is *z*-score-normalized counts for each gene.

**Extended Data Fig. 4:**
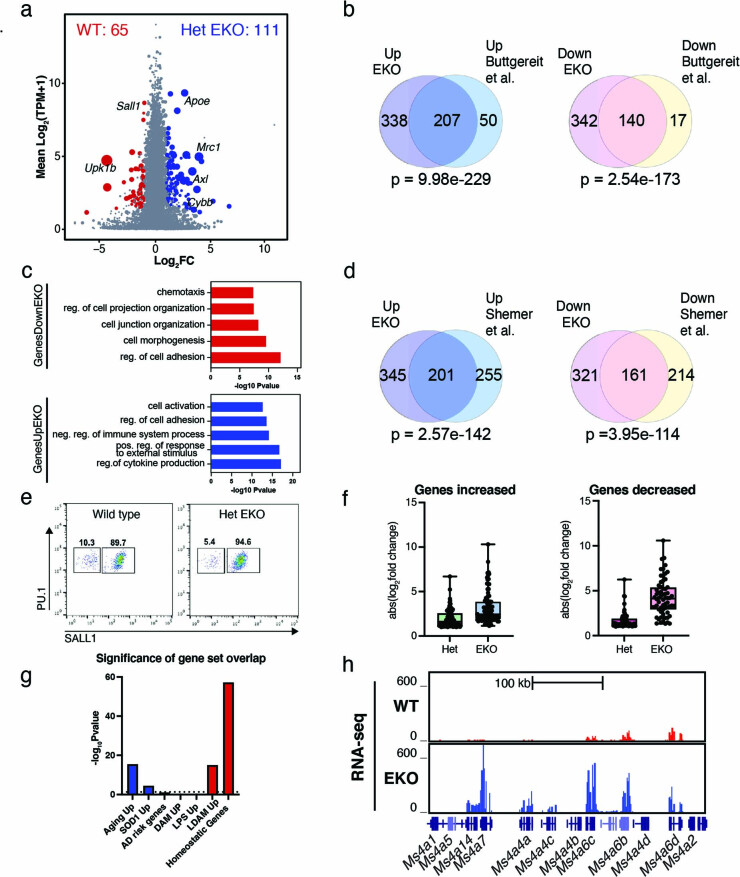
Transcriptional changes in *Sall1* EKO and Het EKO microglia. **a**. MA plot of RNA-seq data from WT versus Het EKO microglia. n = 3/genotype. **b**. Overlap of differential genes (p-adj. < 0.05 and log_2_FC > 1, from DESeq2 analysis (Wald’s test with multiple testing correction using Benjamini–Hochberg method)) identified in EKO microglia vs WT and *Sall1* conditional knockout versus control mouse microglia from Buttgereit et al.^3^. P-values for overlaps were calculated using one-tailed Fisher exact test. **c**. Metascape GO analysis of pathways significantly changed in EKO vs WT microglia. **d**. Overlap of differential genes (p-adj. < 0.05 and log_2_FC > 1, from DESeq2 analysis (Wald’s test with multiple testing correction using Benjamini–Hochberg method)) identified in EKO vs WT microglia and engrafted vs. endogenous microglia (Shemer et al., 2018)^25^. P-values for overlaps calculated using one-tailed Fisher exact test. **e**. Flow cytometry of WT and Het EKO brain nuclei stained for PU1 and SALL1. **f**. Boxplot of log_2_FC of differential genes shared between Het and EKO microglia (n = 3/genotype). Median (center line), whiskers (max, min), box edges (25th - 75th percentile). **g**. Significance of gene set overlaps from Fig. 2g. P-values were calculated using one-tailed Fisher exact test for the overlap between differentially expressed genes in the Sall1 EKO vs differentially expressed genes in aging^29^, the SOD model of ALS^29^, AD risk genes^32^, upregulated genes in Disease Associated Microglia (DAM)^30^, genes upregulated in LPS^29^, genes upregulated in lipid droplet associated microglia (LDAMs)^31^ and microglia homeostatic genes^10,11,40^. Dotted line represents p-value = 0.05. **h**. Expression of *Ms4* family genes in EKO and WT microglia.

Analysis of transcriptomes of WT, Het EKO and EKO microglia revealed progressive changes in microglia gene expression that correlated with the changing levels of *Sall1* (Fig. 2a and Extended Data Fig. 4a). Nearly all genes observed to be differentially regulated in Het EKO microglia are contained in the sets of differentially regulated genes in EKO microglia (Fig. 2b). Differentially regulated genes in EKO microglia also overlapped with the majority of genes observed to be differentially expressed following deletion of *Sall1* in mature mice using a conditional Cre recombinase expressed under the control of the *Sall1* locus itself^3^ (Extended Data Fig. 4b). Upregulated genes are significantly enriched for terms related to cytokine production, response to external stimuli, and regulation of immune system processes (Fig. 2c and Extended Data Fig. 4c), while downregulated genes are associated with processes including cell adhesion, cell morphogenesis and cell junction organization (Fig. 2d and Extended Data Fig. 4c).

We defined a set of 328 highly specific microglia signature genes based on a >10-fold higher level of expression in microglia compared with their average expression across 7 different macrophage subtypes using data derived from consistent methods for macrophage isolation and library preparation^11,23,24^. Notably, in this comparison, *Sall1* is the most differentially expressed mRNA (Supplementary Table 1). Of these microglia signature genes, 108 were among the 482 genes downregulated >2-fold in the EKO, whereas only 6 overlapped with the 544 genes upregulated >2-fold in the EKO (Fisher’s exact test *P* value = 1.49 × 10^−63^ and 0.99, respectively, Fig. 2e). We considered the possibility that some of these changes in gene expression could be due to loss of yolk sac-derived microglia and replacement by hematopoietic stem cell (HSC)-derived cells. Several independent studies documented that HSC-derived cells that engraft the brain following depletion of embryonically derived microglia do not express *Sall1* even after long residence times in the brain^25–27^. These cells exhibit substantial differences in gene expression compared with yolk sac-derived microglia, including some differences that are observed in Het EKO and EKO microglia (Extended Data Fig. 4d). However, HSC-derived cells cannot explain the altered pattern of gene expression in Het EKO microglia, because ~95% of the microglia sorted for gene expression express *Sall1*, albeit at ~50% of the level of WT microglia (Fig. 1c and Extended Data Fig. 4e) and are thus of embryonic origin. Nearly all the genes differentially regulated in Het EKO are contained within the set of differentially regulated genes in EKO microglia but are more highly differentially expressed in EKO microglia (Extended Data Fig. 4f), consistent with progressive loss of function of *Sall1* in embryonically derived cells. In addition, there are differences in the patterns of gene expression of Het EKO and EKO microglia with HSC-derived cells that engraft the brain that are incompatible with substantial replacement of yolk sac-derived microglia. For example, *Sall3* is a member of the SALL TF family that, like *Sall1*, is expressed in yolk sac-derived microglia but not at all in HSC-derived cells^25–27^. *Sall3* expression is unchanged in Het EKO and EKO microglia (Fig. 2f), which is inconsistent with major replacement by HSC-derived cells. Conversely, HSC-derived cells express numerous genes that are not expressed by yolk sac-derived microglia, including *Ccr2* and *Lgals3*, the latter of which has recently been described as a long-lasting marker of HSC-derived cells that engraft the brain^28^. *Ccr2* and *Lgals3* are not expressed in WT, Het EKO or EKO microglia as isolated for these studies (Fig. 2f). Lastly, gene expression changes in EKO microglia are largely concordant with changes resulting from conditional deletion of *Sall1* in adult mice (Extended Data Fig. 4b). In concert, these analyses are most consistent with Het EKO and EKO microglia being of embryonic origin, although fate mapping studies would be required to definitively answer this question.

Recent studies have identified a spectrum of microglial phenotypes across multiple mouse models and disease states. We compared EKO gene expression (adjusted *P* value (p-adj) <0.05) with previously published transcriptomic profiles from microglia in the context of aging, microglia from the SOD1 model of amyotrophic lateral sclerosis (ALS)^29^, microglia from mice after acute peripheral lipopolysaccharide (LPS) treatment^29^, disease-associated microglia (DAM) identified in the 5xFAD mouse model of Alzheimer’s disease^30^, lipid droplet accumulating microglia (LDAM) identified in aging^30,31^ and mouse homologs of Alzheimer’s disease risk loci^32^ with the EKO gene signature, finding significant associations for each comparison (Fig. 2g and Extended Data Fig. 4g), and suggesting that quantitative reductions in *SALL1* expression during aging or disease could contribute to pathogenic microglia phenotypes.

### Genomic sequence determinants of SALL1 binding

**Fig. 3:**
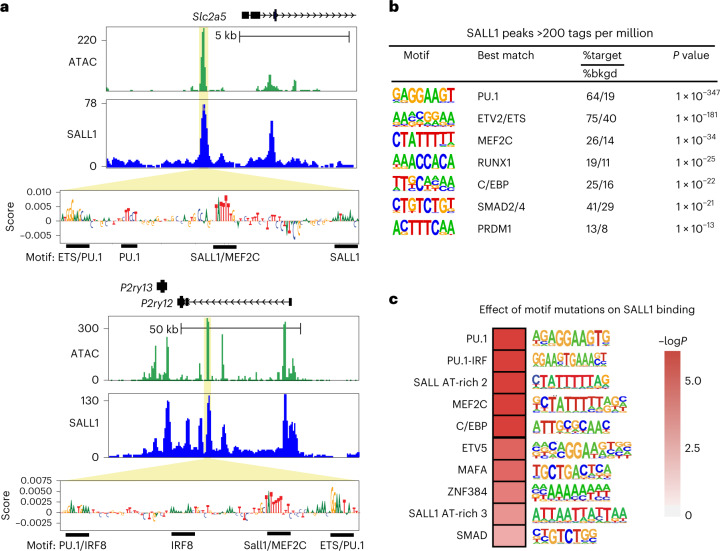
Determinants of SALL1 DNA binding in microglia. **a**, Genome browser images of SALL1 binding sites in regions of open chromatin in the vicinity of the *Slc2a5* (top) and *P2ry12* (bottom) genes that are positively regulated by *Sall1*. Panels below the browser tracks represent nucleotide importance scores defined by a machine learning model trained to predict SALL1 tag counts. Clusters of sequences with high importance scores that show similarity to TF motifs are underlined. See also Extended Data Figs. 6 and 7. **b**, De novo motif analysis of SALL1 peaks containing >200 tags per million at regions of open chromatin. %target is number of target sequences with motif over total target sequences; %bkgd (%background) is the number of background sequences with motif over total background sequences. *P* values calculated using binomial distribution in HOMER. **c**, Effects of natural genetic variation on SALL1 binding in microglia derived from C57BL/6J, PWK and SPRET mice. The heat map represents the corrected *P* values for the test of whether a SNP or InDel in the indicated motif results in a strain-specific reduction in SALL1 binding. *P* values were based on Wilcoxon signed-rank two-sided tests after Benjamini–Hochberg procedure to correct for multiple comparisons. See also Extended Data Fig. 7.

**Extended Data Fig. 5:**
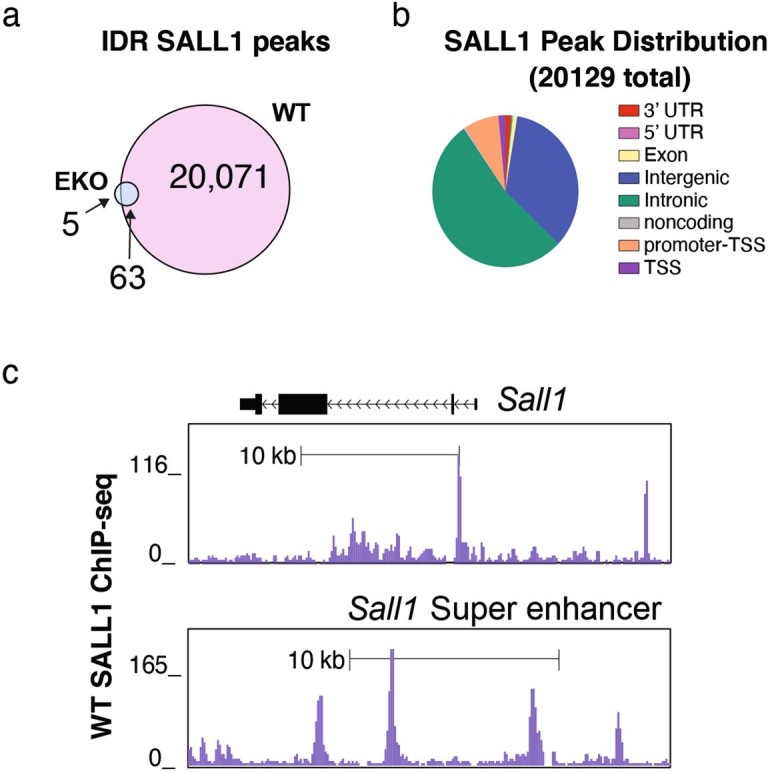
SALL1 ChIP-seq in mouse microglia. **a**. Overlap of WT and EKO SALL1 IDR ChIP peaks. **b**. Genomic distribution of SALL1 peaks. **c**. Genome browser tracks of WT SALL1 ChIP signal at the *Sall1* promoter and super enhancer.

Despite substantial evidence pointing to SALL1 as an essential regulator of microglia identity, little is known about the genes that SALL1 may directly regulate or the underlying mechanisms. To address these questions, we performed ChIP–seq for SALL1 in sorted SALL1^+^/PU1^+^ nuclei (Supplementary Material 1). We defined 20,139 reproducible SALL1 peaks in WT microglia, whereas ChIP–seq for SALL1 in EKO microglia recovered fewer than 70 reproducible peaks (Extended Data Fig. 5a). The majority of SALL1 binding sites localized to intronic and intergenic regions, with a small portion of peaks falling within TSS-promoter regions (Extended Data Fig. 5b), including the *Sall1* promoter and enhancer itself (Extended Data Fig. 5c). SALL1 was also observed to bind at key microglia genes, such as *Slc2a5* and *P2ry12* at sites of open chromatin (Fig. 3a).

De novo motif enrichment analysis of the most confident SALL1 peaks (>200 tag counts per million/peak = 1,620 peaks) recovered motifs recognized by microglia lineage determining factors, including PU.1, PU.1/IRF ternary complexes, and members of the MEF, RUNX, C/EBP and SMAD families of TFs (Fig. 3b). A consensus SALL1 motif has not been established, but prior studies demonstrated that SALL1 interacts with AT-rich sequences^22^, and recent crystallography studies of the conserved Zn finger domains of SALL4 revealed the structural basis for recognition of the consensus sequence AATA within the context of an extended A/T-rich sequence^33^. Of interest, the inverse complement of AATA (TATT) is present in the 5′ end of the enriched motif assigned to MEF2C (Fig. 3b), which is overall AT-rich and matches sequences previously shown to directly bind SALL1.

**Extended Data Fig. 6:**
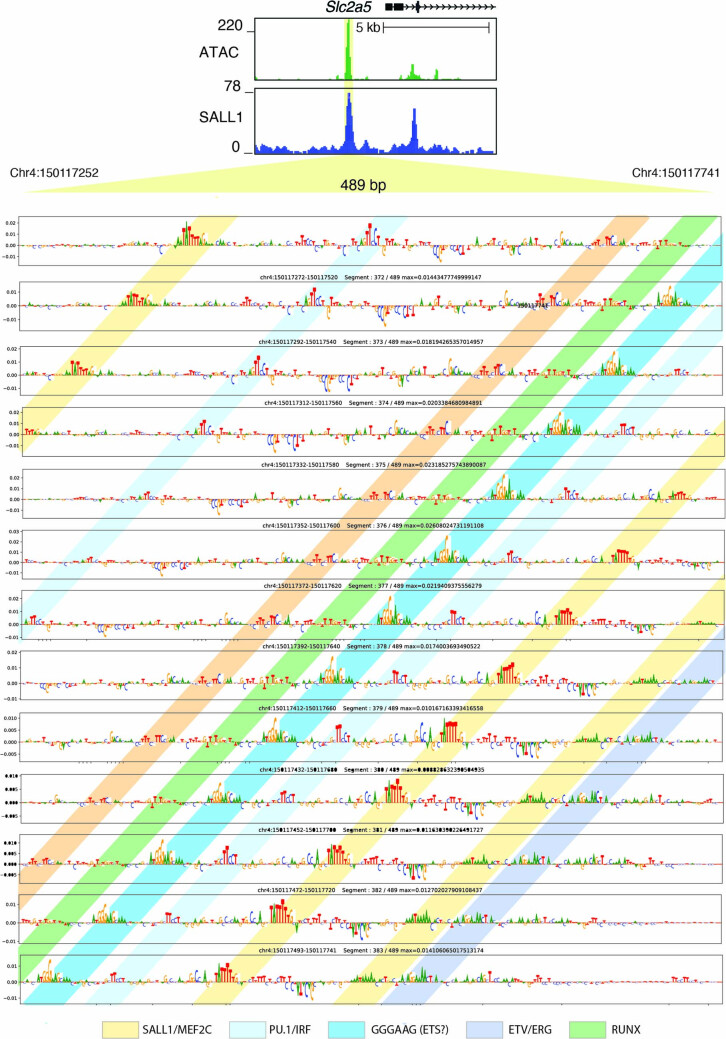
Nucleotide importance scores determined by a machine learning model trained to predict SALL1 tag counts. The tracks represent importance scores calculated for tiled 250 bp sequences at 20 bp increments of the indicated 489 bp region of the putative *Sall1* enhancer upstream of *Slc2a5* depicted in Fig. 3a. Diagonal stripes highlight blocks of important nucleotides corresponding to TF motifs.

To gain further insight into sequence determinants of SALL1 binding, we implemented the convolutional neural network framework of DeepSTARR^34^. DNA segments were subselected from within ATAC peaks to construct the training dataset. Post model training, we derived nucleotide contribution scores for specific DNA elements using DeepLIFT^35^. The model associated high scores with clusters of nucleotides corresponding to AT-rich sequences containing a TATT motif in addition to nearby clusters corresponding to motifs recognized by PU.1, C/EBP and SMAD factors, among others, suggesting the configurations of these motifs driving the prediction of high SALL1 tag counts. Examples of the output of this analysis are provided for regions within putative enhancers below SALL1 peaks present at putative regulatory elements in the *Slc2a5* and *P2ry12* genes (Fig. 3a). Nucleotide importance scores for the entire region of open chromatin of *Slc2a5* are shown in Extended Data Fig. 6.

**Extended Data Fig. 7:**
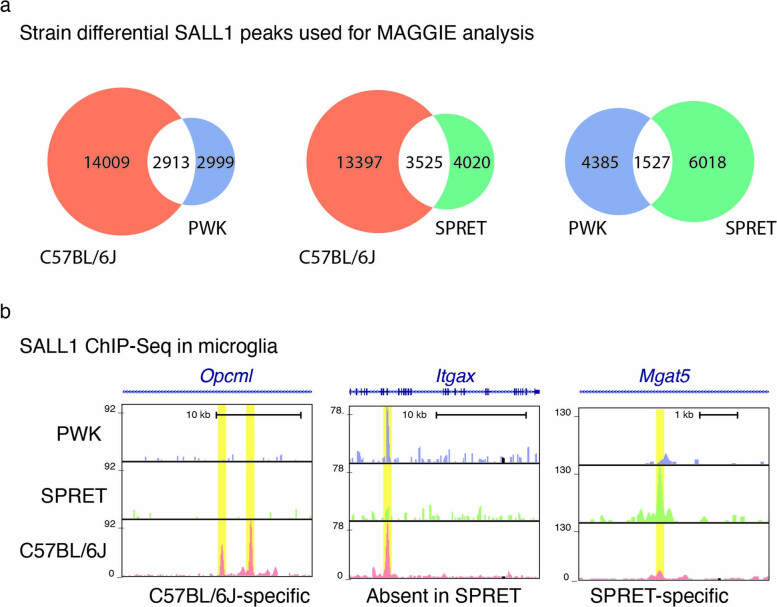
Strain-specific differences in SALL1 binding. **a**. Quantification of similar and strain-preferential SALL1 peaks in pair-wise comparisons of peaks defined by ChIP-seq for SALL1 in microglia derived from C57BL/6 J, PWK and SPRET mice. **b**. Representative examples of SALL1 peaks exhibiting variation between strains at the indicated genomic locations.

As a second independent and confirmatory approach, we investigated the impact of the ~40 million single nucleotide polymorphisms (SNPs) and InDels that distinguish C57BL/6J mice from PWK and SPRET mice on the binding of SALL1. ChIP–seq of SALL1 in microglia derived from PWK and SPRET mice identified more than 40,000 SALL1 strain-specific peaks (Extended Data Fig. 7a,b). We then systematically interrogated strain-specific SALL1 peaks for the frequency of mutations in TF recognition motifs using the motif mutation analysis tool MAGGIE. MAGGIE associates changes of epigenomic features at homologous sequences with motif mutations caused by genetic variation to prioritize motifs that probably contribute to the strain-specific difference^36^. We included all motifs derived from literature sources^22,33^ and de novo motif enrichment analysis (for example, SALL1 AT-rich 2 and SALL AT-rich 3, Fig. 3c). This analysis identified more than a dozen motif clusters in which motif mutations were significantly associated with strain differential SALL1 binding, the top ten of which are shown in Fig. 3c. Mutations in PU.1 and PU.1/IRF motifs had the most significant effects, consistent with an essential role of PU.1 as a pioneer TF required for SALL1 binding and the presence of these motifs in a high fraction of SALL1 peaks. Notably, mutations in the MEF motif containing the AATA core SALL1 recognition motif had the third most significant effects. In concert with the recently established structural determinants of DNA binding by the paired Zn fingers of SALL TFs, and the results of machine learning analyses, these findings are thus most consistent with the MEF recognition motif also mediating direct DNA binding of SALL1.

### SALL1 functions as a repressor and activator in microglia

**Fig. 4:**
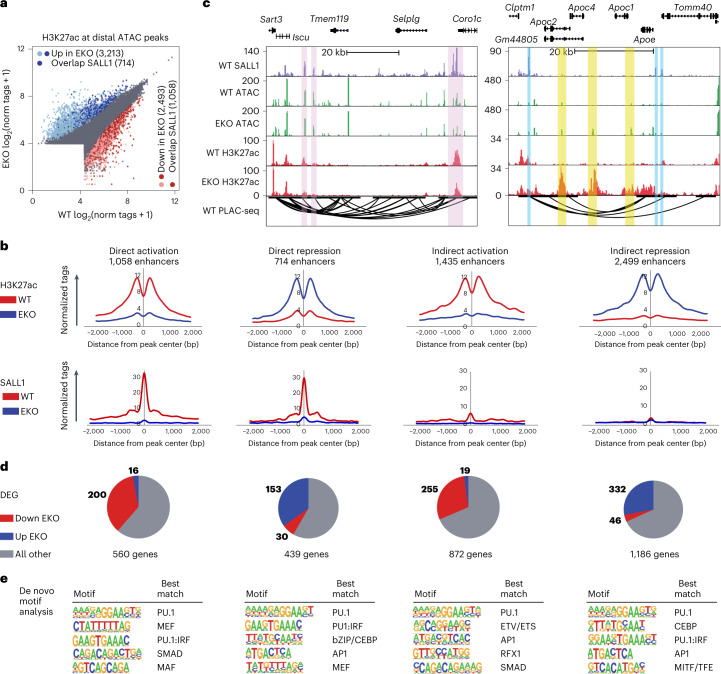
SALL1 is both an activator and repressor in microglia. **a**, Scatter plot of distal ATAC-associated H3K27ac overlapping with SALL1 binding sites. ATAC: *n* = 5 per group; H3K27ac: *n* = 2 per group. Color codes indicate significant changes (light-red and light-blue are p-adj <0.05, FC >2 or <−2, calculated from DESeq2 analysis (Wald’s test with multiple testing correction using Benjamini–Hochberg method)) and significant changes overlapping with SALL1 binding sites (dark red and dark blue). See also Extended Data Fig. 8. **b**, Histograms of normalized H3K27ac and SALL1 counts from EKO and WT microglia at peak subsets defined in **c**. Red, WT; blue, EKO. **c**, Genome browser tracks of SALL1 binding, ATAC, H3K27ac, p300 and PLAC-seq in WT microglia, and ATAC, H3K27ac and p300 in EKO microglia at indicated genes. Pink highlights indicate regions PLAC-connected to promoters where SALL1 binds in WT and loses H3K27ac/p300 signal in EKO microglia. Blue highlights indicate regions where SALL1 binds in regions PLAC-connected to promoters, and yellow highlights indicate regions with an absence of SALL1 binding and increased H3K27ac/p300 signal in EKO microglia. **d**, Overlap of genes nearest to each H3K27ac subset and genes differentially expressed in EKO microglia as compared with WT microglia (p-adj <0.05, calculated from DESeq2 analysis (Wald’s test with multiple testing correction using Benjamini–Hochberg method). **e**, Enriched motifs in each subset of differential distal chromatin regions using GC-matched genomic background. See also Extended Data Fig. 8.

**Extended Data Fig. 8:**
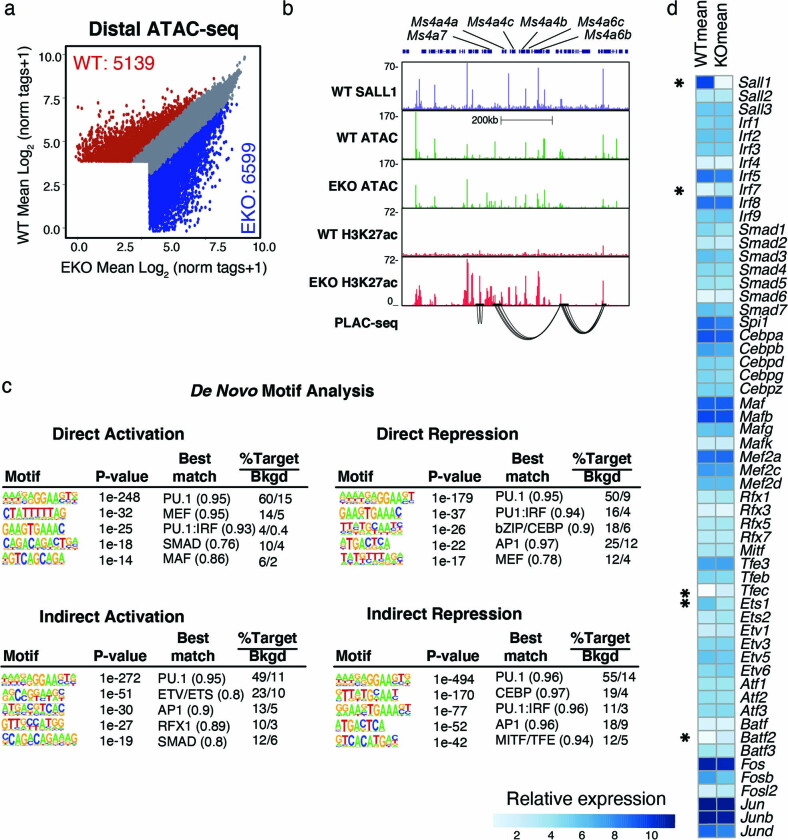
SALL1 ChIP and changes in the chromatin landscape of EKO microglia. **a**. Scatterplot of ATAC peaks in WT vs EKO. n = 5/group. Color codes indicate significant changes (dark red and dark blue are p-adj < 0.05, log_2_FC > 1, from DESeq2 analysis (Wald’s test with multiple testing correction using Benjamini–Hochberg method)). **b**. Genome browser of WT SALL1 ChIP and ATAC/H3K27ac ChIP in WT and EKO microglia at the Ms4 locus. **c**. Statistics associated with motifs illustrated in Fig. 4 (calculated from binomial distribution using HOMER). **d**. Heatmap of expression of TFs identified in the motif analysis in Fig. 4e in EKO and WT microglia. Stars indicate genes with expression changes log_2_FC > 1 or <-1 and p-adj. < 0.05 in EKO microglia, from DESeq2 analysis (Wald’s test with multiple testing correction using Benjamini–Hochberg method).

To link the genomic binding of SALL1 to its transcriptional functions, we performed ATAC-seq and H3K27ac ChIP–seq in EKO microglia. Analysis of ATAC-seq data from WT and EKO microglia indicated that loss of SALL1 was associated with a >2-fold decrease in ATAC signal at 5,139 distal sites and a >2-fold increase at 6,599 distal sites (p-adj <0.05, Extended Data Fig. 8a). We then annotated every distal ATAC peak (>3,000 bp from TSS) with normalized H3K27ac tags (±500 bp from the peak center) in WT and EKO microglia to identify putative enhancers. Using a cutoff of >16 normalized H3K27ac tags, this analysis captured 38,864 ATAC peaks with features of active enhancers (Fig. 4a). Among this set, 3,213 distal regions exhibited a >2-fold increase in H3K27ac (blue points in Fig. 4a) and 2,493 distal regions exhibited a >2-fold decrease in H3K27ac (red points in Fig. 4a) in EKO microglia (p-adj <0.05) (Fig. 4a). We then intersected the putative enhancers that gained or lost H3K27ac in EKO microglia with SALL1 peaks. This analysis revealed that 714 regions with increased H3K27ac overlapped with at least one SALL1 binding site (22% of total upregulated peaks), while 1,058 regions with decreased H3K27ac overlapped with at least one SALL1 binding site (42% of downregulated peaks) (dark-red and dark-blue points in Fig. 4a). These annotations were used to define four putative classes of enhancers (Fig. 4b): those consistent with direct activation by SALL1 (presence of SALL1 and loss of H3K27ac in EKO *n* = 1,058), those consistent with direct repression by SALL1 (presence of SALL1 and gain of H3K27ac in EKO, *n* = 714), those consistent with indirect activation by SALL1 (lack of SALL1 and loss of H3K27ac in EKO, *n* = 1,435) and those consistent with indirect repression by SALL1 (lack of SALL1 and increase in H3K27ac, *n* = 2,499).

Examples of putative enhancers exhibiting loss of H3K27ac in EKO microglia at sites of SALL1 binding are provided by a genomic region containing the microglia signature genes *Tmem119* and *Selplg* (Fig. 4c). These genes, which are strongly dependent on *Sall1* for expression (Fig. 2a), are located amidst multiple chromatin loops defined by PLAC-seq that connect the *Tmem119* and *Selplg* promoters to SALL1 binding sites (shaded in lavender). A contrasting example is provided by a genomic region containing the *Apoe*, *Apoc1*, *Apoc2*, *Apoc4* and *Gm44805* genes. These genes reside within an active chromatin compartment as defined by Hi-C assays of both WT and EKO microglia but are upregulated from 10-fold to more than 100-fold in EKO microglia. These genes reside within PLAC-seq defined loops that are bounded at each end by SALL1 peaks (Fig. 4c, blue stripes). ATAC-seq and H3K27ac signal do not change at these SALL1 binding sites in the EKO microglia but are markedly increased at multiple enhancer-like locations within the PLAC-seq loops that are not bound by SALL1 (yellow stripes, Fig. 4c), consistent with an indirect mechanism of repression of the genes within this region in WT cells. A similar pattern is observed within the *Ms4a* locus (Extended Data Fig. 8b).

To examine the relationships of changes in H3K27ac and SALL1 at distal regions with microglial gene expression at a genome-wide scale, we identified genes associated with each affected enhancer-like region and overlapped these genes with the EKO gene signature (Fig. 4d). Sites bound by SALL1 that lose H3K27ac in EKO are associated with 560 genes; 200 (36%) of these genes are significantly downregulated in EKO microglia, whereas only 16 (2.8%) are upregulated (p-adj <0.05). Conversely, sites bound by SALL1 that gain H3K27ac are associated with 439 genes, 153 (35%) of which are upregulated in EKO microglia in comparison with 30 (6.8%) that are downregulated. These findings are consistent with SALL1 acting to directly activate or repress gene expression via actions at nearby enhancers. At putative enhancers that gain or lose H3K27ac in EKO that do not contain a SALL1 peak and are indirectly regulated, changes in nearby gene expression are consistent with the corresponding gain or loss of enhancer H3K27ac (Fig. 4d).

We next performed de novo motif analysis of the four classes of differentially regulated enhancers. In all cases, the most highly enriched motif corresponded to the consensus binding site for PU.1, consistent with a major role in the selection of all four classes of microglia regulatory elements^37,38^ (Fig. 4e and Extended Data Fig. 8c). At the 1,058 enhancer-like elements bound by SALL1 exhibiting loss of H3K27ac in EKO microglia, the next most significantly enriched sequence corresponded to a MEF motif that we now show is also recognized by SALL1. The following most significant motifs are a PU.1:IRF composite element, and motifs recognized by SMADs and MAF family members. The presence of SMAD motifs was of particular interest because members of the SMAD TF family mediate transcriptional responses to TGFβ signaling, which is required for microglia development^7,8,39^.

PU.1, PU.1:IRF and MEF motifs were also observed at enhancer-like elements bound by SALL1 exhibiting gain of H3K27ac in EKO microglia (Fig. 4e and Extended Data Fig. 8c). In addition, these regions exhibited preferential enrichment for motifs recognized by C/EBP and AP-1 family members, suggesting that SALL1 might function to directly repress their transcriptional activities at these locations. Peaks decreased in EKO not overlapping with SALL1 were enriched for ETV/ETS, AP1, the RFX family and SMADs, indicating that these factors may be responsible for changes in enhancer activity independent of direct SALL1 binding (Extended Data Fig. 8c). Regions with increased H3K27ac and no overlap with SALL1 binding sites were enriched with motifs for the CEBP family, the PU1:IRF8 heterodimer, the AP1 family and the MITF/TFE family of TFs, suggesting activating roles at these locations. We examined the expression of TFs recognizing motifs identified in the de novo motif analysis and found that *Irf7*, *Tfec* and *Batf2* were significantly upregulated in EKO (fold change (FC) >2, p-adj <0.05) and expression of *Ets1* was significantly decreased in EKO (FC <−2, p-adj <0.05) (Extended Data Fig. 8d).

### SMAD4 and SALL1 regulate a common set of microglia identity genes

**Fig. 5:**
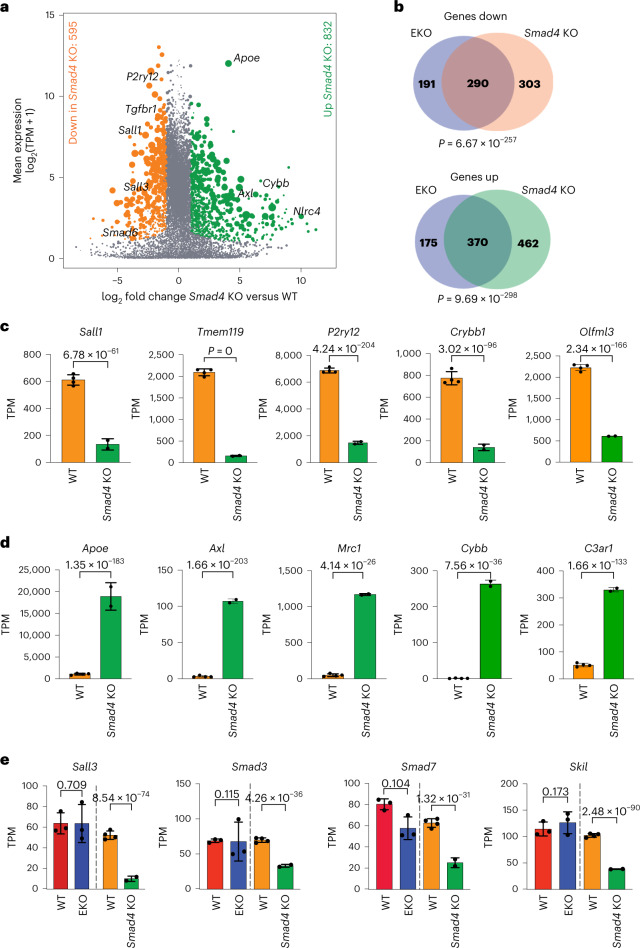
Loss of *Smad4* phenocopies loss of *Sall1*. **a**, MA plot of RNA-seq data comparing WT and Smad4 cKO microglia. *n* = 2–4 per group. DEGs were defined as p-adj <0.05, FC >2 or <−2, and log_2_(TPM + 1) >4 in at least one group. p-adj calculated from DESeq2 analysis (Wald’s test with multiple testing correction using Benjamini–Hochberg method). See also Extended Data Fig. 9. **b**, Overlap of DEGs in EKO microglia versus Smad4 cKO microglia. *P* value was calculated using one-tailed Fisher exact test. **c**, Bar plots for expression of downregulated genes in Smad4 cKO (green) as compared with WT (orange) microglia. *n* = 2–4 per genotype. Data are represented as mean with standard deviation, p-adj from DESeq2 analysis (Wald’s test with multiple testing correction using Benjamini–Hochberg method). **d**, Bar plots for expression of upregulated genes in Smad4 cKO (green) microglia as compared with WT (orange). *n* = 2–4 per genotype. Data are represented as mean with standard deviation, p-adj from DESeq2 analysis (Wald’s test with multiple testing correction using Benjamini–Hochberg method). **e**, Bar plots comparing expression of genes differentially expressed in WT versus EKO and WT versus Smad4 cKO, *n* = 2–4 per genotype. Data are represented as mean with standard deviation, p-adj from DESeq2 analysis (Wald’s test with multiple testing correction using Benjamini–Hochberg method).

**Extended Data Fig. 9:**
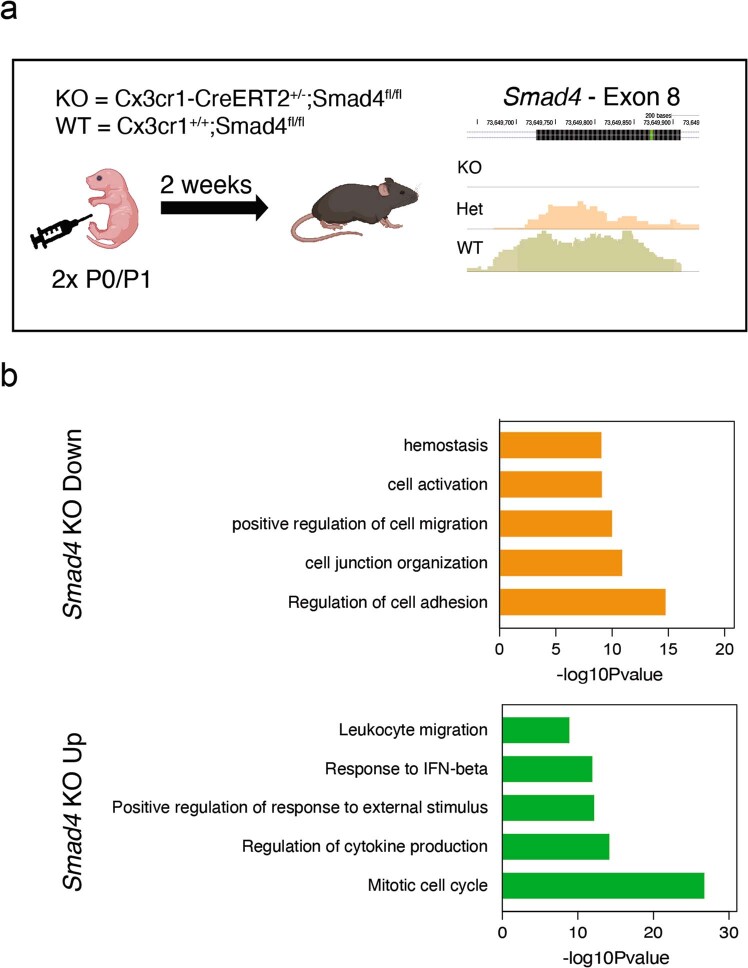
Conditional, inducible deletion of *Smad4* in microglia. **a**. Schematic of experimental setup for conditional Smad4 cKO mice (gen- erated in BioRender.) The indicated genotypes were treated with tamoxifen on days P0 and P1 and microglia were isolated for analysis two weeks later. The inset indicates effective excision of floxed exon 8 following tamoxifen treatment as evidenced by the absence of sequencing tags. **b**. Metascape GO analysis of genes significantly changed in Smad4 cKO microglia. P-values calculated from hypergenometric distribution from Mestascape.

TGFβ signaling, which plays an essential role in establishing microglia identity and promoting microglial survival^8,39^, is known to control expression of *Sall1* and other key microglial genes^8,39–41^. Signaling via TGFBR2 induces the activation of the receptor-associated SMADs (R-SMADs), SMAD2 and SMAD3. These R-SMADS complex with SMAD4 and translocate to the nucleus, where they localize to SMAD-binding elements at TGFβ target genes^42^. The enrichment of SMAD family motifs in the *Sall1* SE and in enhancer-like regions losing H3K27ac in EKO suggested that SMADs may be both controlling *Sall1* expression and playing roles as important binding partners of SALL1 in microglia. Since SMAD4 is a unique co-factor utilized by all receptor activated SMADs, we generated an inducible deletion of *Smad4* in microglia (Cx3cr1^ERT2^ × Smad4^*fl/fl*^, Smad4 cKO, Extended Data Fig. 9a) and measured the effects of *Smad4* cKO on the microglial transcriptome. Smad4 cKO resulted in downregulation of 595 genes and upregulation of 832 genes (FC >2, p-adj <0.05) (Fig. 5a). Genes upregulated in *Smad4* cKO microglia were related to functions including cell cycle, cytokine production, response to external stimulus and leukocyte migration (Extended Data Fig. 9b). Downregulated genes were affiliated with categories such as regulation of cell adhesion, cell junction organization and regulation of cell migration (Extended Data Fig. 9b).

To examine similarities between EKO and Smad4 cKO transcriptional signatures, we overlapped the differentially expressed genes (DEGs) from each condition (Fig. 5b). Sixty percent (290/482) of genes decreased in EKO overlapped with genes decreased in Smad4 cKO (*P* = 6.67 × 10^−257^) (Fig. 5c). Notably, loss of Smad4 also resulted in a 75% decrease in *Sall1* expression (Fig. 5c), consistent with prior studies demonstrating that *Sall1* is positively regulated by TGFβ1 and further suggesting that the Smad4 cKO should partially phenocopy the *Sall1* EKO. Sixty-eight percent (370/545) of genes increased in EKO overlapped significantly with genes increased in Smad4 *c*KO microglia (p-adj 9.69 × 10^−298^), including *Apoe*, *Axl*, *Mrc1*, *Cybb* and *C3ar1* (Fig. 5d). In contrast, loss of *Smad4*, but not *Sall1*, caused a decrease in *Sall3* and members of the TGFβ signaling pathway, such as *Smad3*, *Smad7* and *Skil* (Fig. 5e).

### SALL1 regulates DNA binding and function of SMAD4

**Fig. 6:**
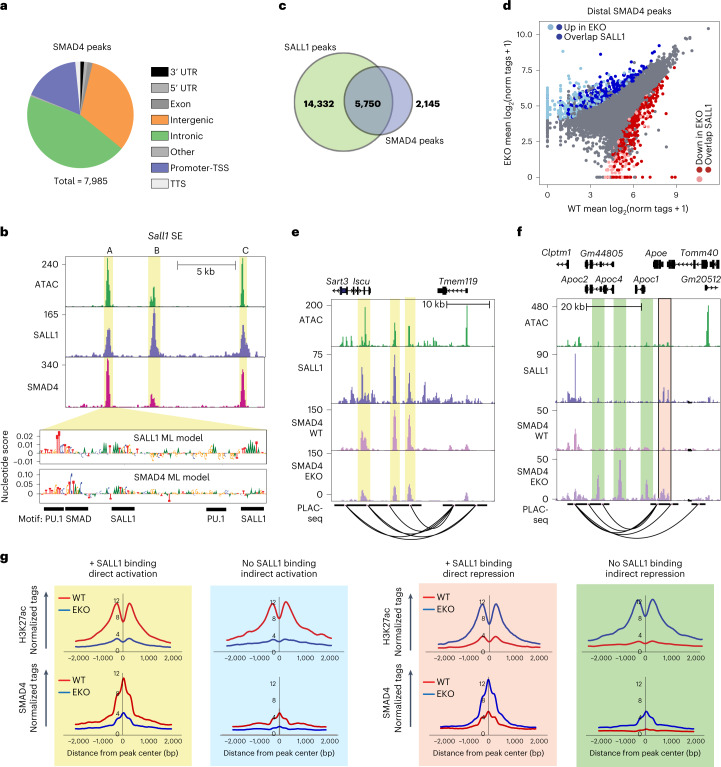
SALL1 enforces a microglia-specific pattern of DNA binding and function of SMAD4. **a**, Pie chart representing distribution of IDR-defined SMAD4 peaks (*n* = 2). UTR, untranslated region. **b**, Genome browser tracks of H3K27ac ChIP–seq, ATAC, and SALL1-, PU.1- and SMAD4-ChIP–seq at the *Sall1* SE in WT microglia. Yellow highlights and A, B and C labels represent the three main regions of open chromatin in the SE. **c**, Overlap of IDR-defined SALL1 and SMAD4 peaks in WT microglia. **d**, Scatter plot of distal SMAD4 peaks overlapping with SALL1 binding sites. Color codes indicate significant changes (light red and light blue are p-adj <0.05, FC >2 or <−2, p-adj from DESeq2 analysis (Wald’s test with multiple testing correction using Benjamini–Hochberg method)) and significant changes overlapping with SALL1 binding sites (dark red and dark blue). **e**, Genome browser tracks of ATAC, SALL1 and PLAC-seq in WT microglia and SMAD4 in EKO and WT microglia at the *Selplg/Tmem119* locus. Yellow highlights indicate regions where SMAD4 binding is diminished in EKO upon loss of SALL1 binding. **f**, Genome browser tracks of ATAC, SALL1 and PLAC-seq in WT microglia and SMAD4 in EKO and WT microglia at the *Apoe* locus. Pink highlight shows region where loss of direct SALL1 binding leads to increased SMAD4 signal in EKO. Green highlights demonstrate regions where SMAD4 binding increases in EKO, independent of a SALL1 binding site. **g**, Histograms of normalized H3K27ac and SMAD4 counts from EKO and WT microglia at peak subsets defined in **c**. Red, WT; blue, EKO.

**Extended Data Fig. 10:**
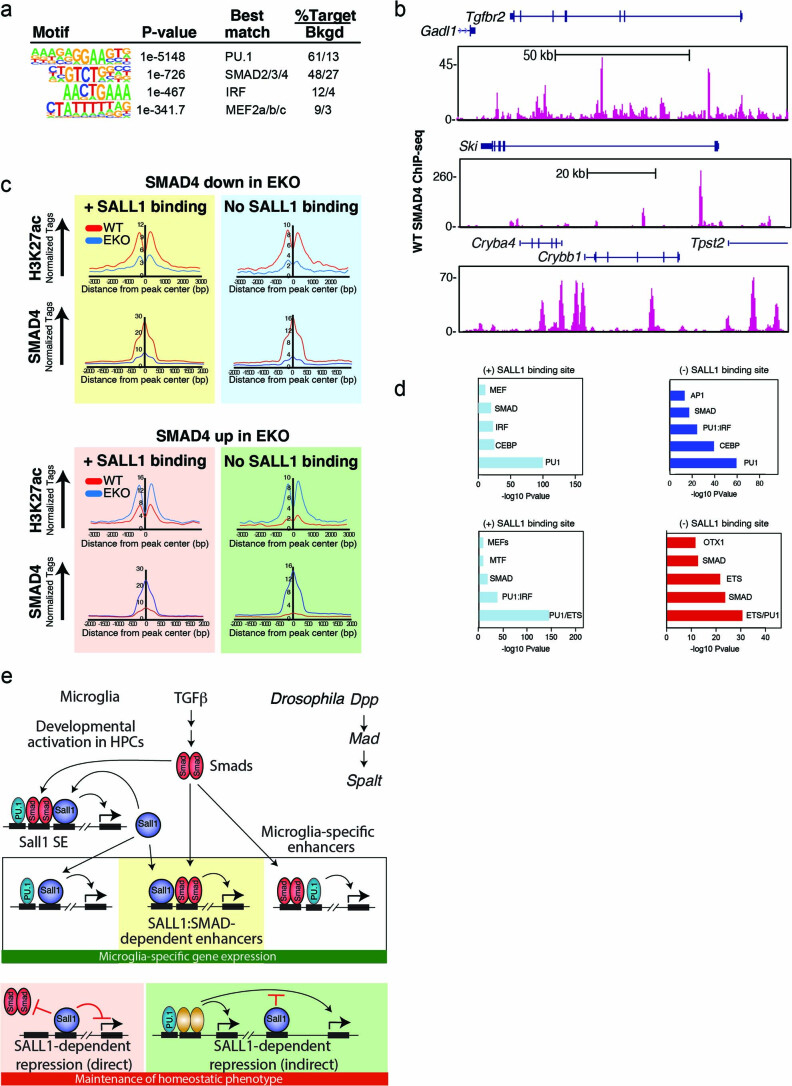
SMAD4 ChIP in WT and EKO microglia. **a**. *De novo* motifs identified in IDR-defined SMAD4 peaks in WT microglia. P-values calculated from binomial distribution using HOMER. **b**. Genome browser of WT SMAD4 binding at microglia genes and TGFβ responsive genes. **c**. Histogram of H3K27ac and SMAD4 signal at differential, distal SMAD4 peaks in EKO vs WT. **d**. *De novo* motif analysis of the SMAD4 peak subsets identified in panel c. P-values calculated from binomial distribution using HOMER. **e**. Schematic of the proposed collaboration between SALL1 and SMAD4 in determining microglia identity.

We next performed ChIP–seq for SMAD4 in sorted microglia nuclei, identifying almost 8,000 peaks, which localized primarily to distal intergenic and intronic regions (Fig. 6a). De novo motif analysis revealed that SMAD4 peaks were enriched for PU.1, SMAD, IRF and AT-rich MEF/SALL1 family motifs, indicating that SMAD4 binding is probably driven by collaborative interactions with microglia lineage determining factors (Extended Data Fig. 10a). As expected, SMAD4 binds to promoters and putative enhancers of genes that are dependent on TGFβ signaling and are associated with microglia identity, such as *Olfml3*, as well as genes encoding known TGFβ pathway regulators, such as *Tgfbr2* and *Ski* (Extended Data Fig. 10b). Notably, SMAD4 binds strongly to regions A, B and C of the *Sall1* SE in close proximity to SALL1 and PU.1 (Fig. 6b), consistent with the presence of conserved SMAD motifs (Extended Data Fig. 1b) and the effects of the Smad4 cKO on *Sall1* expression.

Remarkably, 72% (5750/7985) of SMAD4 peaks overlapped with a SALL1 binding site (Fig. 6c), suggesting that, in addition to roles in the activation of *Sall1* expression, SMADs and SALL1 might also function as collaborative binding partners to regulate microglia-specific enhancers. To probe a potential relationship between SMAD4 and SALL1 binding, we leveraged the lack of SALL1 expression in EKO microglia to assess changes in SMAD4 binding at distal regulatory regions upon loss of SALL1. SMAD4 ChIP–seq in EKO microglia revealed that 645 distal SMAD4 peaks were decreased and 667 distal SMAD4 peaks were increased (FC >2, p-adj <0.05) in comparison with WT microglia (Fig. 6d). Of the SMAD4 peaks that were reduced in EKO microglia, 75% (484/645) overlapped with a SALL1 peak (Fig. 6d), consistent with SALL1 directly contributing to SMAD4 binding at these locations. Reduced SMAD4 binding in the EKO is exemplified at the genomic locus containing *Tmem119* and *Selplg* (Fig. 6e, yellow highlights).

We next used DeepSTARR to train a model predicting SMAD4 binding. Here the SMAD4 motif emerged as the highest-scoring nucleotide group from a list of over 200 sequences that were sorted on the basis of their nucleotide contribution scores. In addition, these regions often contain clusters of high-scoring nucleotides that correspond to MEF2/SALL1 and PU.1 motifs. Nucleotide contribution scores associated with sequences from enhancer element A of the *Sall1* SE are illustrated at the bottom of Fig. 6b. In comparison, the SALL1 model identifies a SALL1/MEF2C motif in the highest-scoring nucleotide group, but also captures nucleotide groups that are related to SMAD and PU.1 motifs (Fig. 6b).

Of the SMAD4 peaks that were gained in EKO microglia, 46% (309/667) overlapped with a SALL1 peak in WT microglia (Fig. 5d). This result suggests that, at these sites, SALL1 functions to directly restrict SMAD4 binding. The 54% of SMAD4 peaks that are gained in EKO microglia and do not overlap with SALL1 peaks provide evidence that the absence of SALL1 also enables redistribution of SMAD4 to alternative locations illustrated by the genomic locus containing *Apoe*, *Apoc1*, *Apoc2*, *Apoc4* and *Gm44805* (Fig. 6f, green highlights).

A global analysis of H3K27ac signal at genomic locations exhibiting gain or loss of SMAD4 found that SMAD4 peaks that increased in EKO microglia, regardless of overlap with a SALL1 binding site, were characterized by an increase in EKO H3K27ac signal (Extended Data Fig. 10c). Conversely, SMAD4 peaks that were downregulated in EKO microglia, regardless of overlap with a SALL1 binding site, were associated with reduced H3K27ac signal (Extended Data Fig. 10c). These results indicate that SMAD4 is primarily acting as an activator of the chromatin landscape at sites that are directly or indirectly affected by SALL1. De novo motif analysis revealed that all subsets of differential SMAD4 peaks shared enrichment for PU1, ETS and SMAD motifs (Extended Data Fig. 10d). SMAD4 peaks gained and lost in EKO that overlapped with a SALL1 binding site were further enriched for AT-rich MEF motifs. In contrast, SMAD4 peaks that were gained in EKO and non-overlapping with SALL1 binding sites were enriched for AP1 motifs (Supplementary Fig. 10d). It is known that SMADs can partner with the AP-1 complex^43,44^ which may indicate that SMAD4 redistribution in EKO is in part driven by collaboration with AP-1.

Lastly, we evaluated SMAD4 binding at each of the four categories of enhancers defined by gain or loss of H3K27ac in EKO microglia and the presence or absence of a SALL1 peak in WT microglia illustrated in Fig. 4b. High levels of SMAD4 binding were observed at enhancers occupied by SALL1 in WT microglia and in which H3K27ac levels fell in EKO microglia (directly activated enhancers). Notably, SMAD4 binding was markedly reduced at these enhancers in EKO microglia (Fig. 6g, top left). Conversely, low levels of SMAD4 binding were observed at enhancers occupied by SALL1 in WT microglia and in which H3K27ac levels increased in EKO microglia (directly repressed enhancers). At these locations, SMAD4 binding increased significantly in EKO microglia (Fig. 6g, bottom left). SMAD4 binding was also observed to decrease at indirectly activated enhancers and increase at indirectly repressed enhancers in EKO microglia, but to a lesser extent than at enhancers bound by SALL1 in WT microglia (Fig. 6g, top and bottom right).

## Discussion

Here we demonstrate that a conserved genomic region 300 kb upstream of the *Sall1* gene functions as a cell-specific SE required for expression of *Sall1* in microglia. The findings that this regulatory region is occupied by SMAD4 and that *Sall1* expression requires TGFβ signaling^39^ are consistent with a model in which TGFβ induces *Sall1* in yolk sac-derived HPCs that enter the embryonic brain by directly activating the *Sall1* SE via SMADs. Furthermore, the genome-wide binding profiles of SALL1 and SMAD4, in concert with epigenetic analyses of WT and EKO microglia, provide strong evidence for an unexpected layer of functional interactions between these two proteins that results in direct activation of hundreds of regulatory elements that are associated with the expression of microglia identity genes (Extended Data Fig. 10e, yellow box). We also find evidence that SALL1 can function as a transcriptional activator independently of SMAD4 and vice versa, probably through collaborative interactions with other microglia lineage determining factors. Collectively, these findings support direct roles of SALL1 and SMADs acting together and independently in the selection and activation of a large fraction of the enhancers that regulate microglia-specific patterns of gene expression.

Remarkably, studies of the homologous *Spalt* gene in *Drosophila* demonstrated that its expression in specific regions of the wing requires the concerted actions of Dpp and Mad, *Drosophila* homologs of TGFβ (refs. ^45–47^) and SMADs. The present finding that SALL1 in turn regulates the DNA binding and function of SMAD4 expands this developmental paradigm to also place SMADs downstream of SALL1. It will be of interest to determine whether the mechanisms by which SALL1 shapes the transcriptional response to TGFβ expanded upon here in microglia may operate in other organ systems in which loss of *Sall1* results in developmental defects.

The observation that hundreds of genes are upregulated in EKO microglia also supports functions of SALL1 as a transcriptional repressor that is required to maintain a microglia-specific and homeostatic phenotype. We observe evidence for both direct and indirect mechanisms of repression. Examples of direct repression are provided by the ~309 SMAD4 peaks that are gained in EKO cells at genomic locations that are occupied by SALL1 in WT microglia. In these cases, SALL1 appears to exert a local repressive function by preventing access of SMADs that would otherwise contribute to enhancer activity (Extended Data Fig. 10e, pink box), thereby restricting the scope of TGFβ/SMAD-dependent gene expression to a microglia-specific pattern. The observation that H3K27ac levels increase at more than 700 SALL1 binding sites in EKO microglia suggests that SALL1 plays similar roles to restrict the binding and function of TFs beyond the family of SMADs. The mechanisms that determine whether SALL1 acts to locally enhance or inhibit SMAD4 binding and functionality represent an important question for future investigation.

In concert, the present studies identify a conserved microglia-specific SE that is activated by SMADs and is required for expression of *Sall1*. Investigation of the genome-wide binding of SALL1 and SMAD4 and the epigenetic consequences of the loss of each protein provides evidence for functional interactions between these proteins that enable TGFβ to induce a microglia-specific program of gene expression. These datasets also represent a substantial new resource for the microglia research community. The finding that haploinsufficiency for *Sall1* is associated with substantial changes in the expression of genes associated with aging and neurodegenerative diseases raises the possibility that quantitative changes in its expression could contribute to disease phenotypes. Among the intriguing and unanswered questions that remain to be solved are why activation of the *Sall1* gene is restricted to HPCs and what are the identities of brain environmental factors required in addition to TGFβ to turn on and maintain *Sall1* expression in microglia. Further studies of the *Sall1* SE are likely to provide insights into these questions.

## Methods

### Mice

All animal procedures were approved by the University of California San Diego Animal Care and Use Committee in accordance with the University of California San Diego research guidelines for the care and use of laboratory animals. The following mice were used in this study: C57BL/6J (The Jackson Laboratory, stock no. 00064), *Sall1* EKO (generated by Glass lab and transgenic core facility, University of California, San Diego), Cx3cr1^CreER^ (ref. ^48^) (The Jackson Laboratory, stock no. 020940), and *Smad4*^*fl/fl*^ (ref. ^49^) (The Jackson Laboratory, stock no. 017462). For experiments with C57BL/6J and *Sall1* Het EKO, EKO, male mice were used between 8 and 12 weeks of age. Experiments for targeted, inducible deletion of *Smad4* were performed on male mice at P0 and microglia were collected at 2 weeks of age. For all experiments, no statistical methods were used to predetermine sample size, but our sample sizes are similar to those reported in previous publications^11,23^. Data distribution was assumed to be normal, but this was not formally tested. Animals were not randomized before tissue collection. Data collection and analysis were not performed blind to the conditions of the experiments. Datasets are from sequential samples for which cell viability and sequencing libraries met technical quality standards.

### Generation of *Sall1* EKO mouse

Sixteen female mice were super-ovulated. Overnight matings were set up, and the following morning the oviducts of each female mouse were collected. Injection of single guide RNAs and Cas9 protein into pronuclei of one-cell-stage zygotes was performed by the UCSD Transgenic Animal Core. Preparation of single guide RNAs was performed as previously described^50^. On the morning of the injection day the reagents were prepared as follows: each CRISPR RNA (protospacers: GAATGACCCTGGCAATCATG, TCCATAAG ATAGCTTAGGGA, CTTGACAGACATTACACAGG, CTAGAATCGGCTTTGGTGCT) was annealed to trans-activating CRISPR RNA in IDTE (10 mM Tris, 0.1 mM EDTA) (pH 7.5) at 95 °C for 5 min ramped down to 25 °C at 5 °C per minute. Cas9 protein (NEB#M0646T) was diluted in IDTE (pH 7.5) and incubated with annealed guide RNAs for 10 min at 22 °C. ssODN (single-stranded oligodeoxynucleotides) and IDTE were then mixed incubated at 22 °C for another 5 min, and spun at 10,000 r.p.m. for 1 min. The supernatant was transferred to a new tube and transferred to the UCSD Transgenic Core for injection. Genetically targeted mice from the CRISPR-mediated deletion were screened by PCR with KOD Xtreme Hot Start DNA polymerase (EMD Millipore) using three primers: 5′F (GGAGAGTGTTCT GGAAAGCAGGGAGA), 5′R internal to the deletion (CTGGCATCTGGAGT CCCAGACACT) and 3′R (GCCCAAAGTTCAAAGACC TGCTGT). 5′F + 5′R internal amplified a 582 bp band from the WT allele and no band from the EKO allele. 5′F and 3′R amplified a 431 bp band from the EKO allele and no band from the WT allele. *Sall1* EKO mice were crossed to C57BL/6J WT mice for at least three generations.

### Tamoxifen-mediated deletion of *Smad4*

Cx3cr1^CreER^ mice were crossed to *Smad4*^*fl/fl*^ mice to generate Cx3cr1^CreER^ *Smad4*^*fl/fl*^ mice. Mice were treated twice with tamoxifen: 75 μg at P0 and 50 μg at P1, and microglia were collected 14 days later.

### Flow cytometry to sort live microglia

Mouse brains were homogenized as previously described^10,11^ by gentle mechanical dissociation. Cells were then incubated in staining buffer on ice with anti-CD16/32 blocking antibody (BioLegend 101319, 1:500) for 15 min, and then with anti-mouse anti-CD11b-APC (BioLegend 101212, 1:100), anti-CD45-Alexa488 (BioLegend 103122, 1:100), and anti-CX3CR1-PE (BioLegend 149006, 1:100) for 25 min. Cell preparations for H3K27ac ChIP–seq, PLAC-seq and Hi-C were fixed with 1% formaldehyde for 10 min and quenched with 0.125 M glycine for 5 min after staining, and subsequently washed three times. Cells were washed once and filtered through a 40 μM cell strainer. Sorting was performed on a Sony MA900 or MoFlo Astrios EQ cell sorter. Microglia were defined as events that were DAPI negative, singlets and CD11b^+^CD45^low^CX3CR1^+^. Isolated microglia were then processed according to protocols for RNA-seq, ATAC-seq and ChIP–seq, Hi-C and PLAC-seq.

### Immunostaining for SALL1 and IBA1

Eight-week-old female WT and *Sall1* EKO mice were perfused with 2% paraformaldehyde, and then the brains were collected and fixed in 4% paraformaldehyde in phosphate-buffered saline (PBS) overnight at 4 °C. After fixation, the brains were washed three times in PBS and cryoprotected in 30% sucrose and embedded in Neg-50 (Epredia) for subsequent cryosection. Then 20 μm sections were cut on cryostat, mounted on Superfrost plus slides (Thermo Scientific, Menzel-Glaser), dried at 37 °C and subjected to immunofluorescence staining. For immunofluorescence, sections were rehydrated, rinsed in PBS for three times, 5 min each. Sections were permeabilized in 0.3% Triton X-100 in PBS and blocked in blocking solution (5% normal donkey serum in PBST) in a humidified chamber for 1 h at 22 °C. Slides were then incubated with the appropriate primary antibodies diluted in blocking solution at 4 °C overnight. The primary antibodies were rat anti-Sall1 (Thermo Fisher, Clone NRNSTNX, 14-9729-82), and rabbit anti-IBA1 (FujiFilm, 019-19741). The next day, sections were washed three times (10 min each) in PBST, incubated with appropriate fluorophore-conjugated secondary antibodies (donkey anti-rat 555, Invitrogen SA5-10027; donkey anti-rabbit 488, Invitrogen R37118) diluted in blocking solution at 22 °C for 2 h, washed three times (10 min each) in PBST, counter-stained with DAPI for 10 min, rinsed once in PBS and mounted with Prolong Gold antifade reagent (Invitrogen, P36931) and imaged on a Nikon Sterling Spinning Disk Confocal Microscope with 60× object images were processed with ImageJ (version 1.53j) (ref. ^51^).

### Sorting crosslinked brain nuclei

Brain nuclei were isolated as previously described^13,18^, with initial homogenization performed with either 1% formaldehyde in Dulbecco’s phosphate-buffered saline or 2 mM DSG (disuccinimidyl glutarate) (ProteoChem) in Dulbecco’s phosphate-buffered saline. Nuclei were stained overnight with PU.1-PE (Cell Signaling 81886S, 1:100), OLIG2-AF488 (Abcam 225099, 1:2,500) or SALL1 AF647 (Thermo Fisher, clone NRNSTNX 51-9279-82, 1:100) or NEUN-AF488 (Millipore MAB 377X, 1:500). Nuclei were washed the following day with 4 ml FACS buffer, passed through a 40 µM strainer, and stained with 0.5 μg ml^−1^ DAPI. Nuclei for each cell type were sorted with a Beckman Coulter MoFlo Astrio EQ cell sorter and pelleted at 1,600*g* for 5 min at 4 °C in FACS buffer. Nuclei pellets were snap frozen and stored at −80 °C before library preparation.

### ATAC-seq library preparation

ATAC-seq libraries were prepared as previously described^18,23,52,53^ with approximately 50,000 sorted microglia. Cells were lysed in 150 µl lysis buffer (10 mM Tris–HCl pH 7.5, 10 mM NaCl, 3 mM MgCl_2_ and 0.1% IGEPAL CA-630 in water). Resulting nuclei were centrifuged at 500*g* for 10 min. Pelleted nuclei were resuspended in 50 μl transposase reaction mix (1× Tagment DNA Buffer (Illumina 15027866) and 2.5 μl DNA enzyme I (Illumina 15027865)) and incubated at 37 °C for 30 min. DNA was purified with Zymo ChIP DNA concentrator columns (Zymo Research D5205), eludated with 11 µl of elution buffer, and amplified using NebNext High-Fidelity 2× PCR Master Mix (New England BioLabs M0541) with the Nextera primer Ad1 (1.25 µM) and a unique Ad2.n barcoding primer (1.25 µM) for 8–12 cycles. Resulting libraries were size selected by gel excision to 155–250 bp, purified and single end sequenced using a HiSeq 4000 (Illumina) for 51 cycles according to the manufacturer’s instructions.

### RNA-seq library preparation

RNA-seq libraries were prepared as previously described^23^ with approximately 100,000 sorted live microglia. FACS-sorted cells were stored in TRIzol LS. Total RNA was extracted from homogenates and cells using the Direct-zol RNA MicroPrep Kit (Zymo Research R2052) and stored at −80 °C until RNA-seq library preparation. mRNAs were enriched by incubation with Oligo d(T) magnetic beads (NEB, S1419S) in 2× DTBB buffer (20 mM Tris–HCl pH 7.5, 1 M LiCl, 2 mM ethylenediaminetetraacetic acid (EDTA), 1% lithium dodecyl sulfate and 0.1% Triton X-100) at 65 °C for 2 min and were incubated at 22 °C while rotating for 15 min. The beads were then washed 1× with RNA Wash Buffer 1 (10 mM Tris–HCl pH 7.5, 0.15 M LiCl, 1 mM EDTA, 0,1% lithium dodecyl sulfate and 0.1% Triton X-100) and 1× with RNA Wash Buffer 3 (10 mM Tris–HCl pH 7.5, 0.15 M NaCl and 1 mM EDTA) before elution in RNA Elution Buffer (10 mM Tris–HCl pH 7.5 and 1 mM EDTA) at 80 °C for 2 min. PolyA selection was performed a second time, and samples were washed 1× with Wash Buffer 1, 1× with Wash Buffer 3 and 1× with 1× SuperScript III first-strand buffer. Beads were then resuspended in 10 µl 2× SuperScript III buffer plus 10 mM dithiothreitol (DTT), and RNA was fragmented at 94 °C for 9 min and immediately chilled on ice before the next step. For first-strand synthesis, 10 µl of fragmented mRNA, 0.5 µl random primers (50 µM) (Thermo Fisher), 0.5 µl SUPERase-In (Ambion), 1 µl dNTPs (10 mM) and 1 µl of DTT (10 mM) were heated for 50 °C for 1 min. At the end of incubation, 5.8 µl water, 1 µl DTT (100 mM), 0.1 µl actinomycin D (2 µg µl^−1^), 0.2 µl of 1% Tween-20 (Sigma) and 0.5 µl of SuperScript III (Thermo Fisher Scientific) were added and incubated in a PCR machine using the following conditions: 25 °C for 10 min, 50 °C for 50 min and a 4 °C hold. The product was then purified with RNAClean XP beads (Beckman Coulter) according to manufacturer’s instruction and eluted with 10 µl nuclease-free water. The RNA/cDNA double-stranded hybrid was then added to 1.5 µl Blue Buffer (Enzymatics), 1.1 µl of dUTP mix (10 mM dATP, dCTP and dGTP and 20 mM dUTP), 0.2 µl RNase H (5 U µl^−1^), 1.05 µl of water, 1 µl of DNA polymerase I (Enzymatics) and 0.15 µl of 1% Tween-20. The mixture was incubated at 16 °C overnight. The following day, the dUTP-marked double-stranded DNA (dsDNA) was purified using 28 µl of SpeedBeads (GE Healthcare), diluted with 20% PEG8000, 2.5 M NaCl to a final concentration of 13% PEG, and eluted with 40 µl elution buffer (DNA elution buffer from Zymo ChIP Clean and Concentrator Kit). The purified dsDNA underwent end repair by blunting, A-tailing and adaptor ligation as previously described^54^ using barcoded adapters (NEXTflex, Bioo Scientific). Libraries were PCR amplified for 16 cycles, size for 200–500 bp size range, quantified using a Qubit dsDNA HS Assay Kit (Thermo Fisher Scientific) and sequenced on a HiSeq 4000 for 51 cycles according to the manufacturer’s instructions.

### ChIP–seq library preparation

Chromatin immunoprecipitation was performed as previously described^55,56^. For H3K27ac ChIP, 500,000–1,000,000 fixed sorted cells or nuclei were thawed on ice and resuspended in ice-cold LB3 (10 mM Tris–HCl pH 7.5, 100 mM NaCl, 1 mM EDTA, 0.5 mM egtazic acid (EGTA), 0.1% Na-deoxycholate and 0.5% *N*-lauroylsarcosine), 1× protease inhibitor cocktail (Sigma). Chromatin was sheared by sonication. Samples were sonicated in a 96-place microtube rack (Covaris cat. no. 500282) using a Covaris E220 for 12 cycles with the following setting: time 60 s, duty cycle 5.0, PIP 175, cycles, 200, amplitude 0.0, velocity 0.0, dwell 0.0. Samples were recovered and spun down at maximum speed, 4 °C for 10 min. The supernatant was then diluted 1.1-fold with ice-cold 10% Triton X-100. One percent of the lysate was kept as ChIP input. Then 25 µl of Dynabeads Protein A was added per sample, in addition to 1 µg of a specific antibody for H3K27ac (Active Motif 39685). The samples were rotated overnight at 4 °C and were washed as follows the next day: 3× with Wash Buffer I (20 mM Tris–HCl pH 7.5, 150 mM NaCl, 2 mM EDTA, 0.1% SDS and 1% Triton X-100) + protease inhibitor cocktail, 3× with Wash Buffer III (10 mM Tris–HCl pH 7.5, 250 mM LiCl, 1% Triton X-100, 1 mM EDTA and 0.7% sodium deoxycholate) + protease inhibitor cocktail, 2× with TET (0.2% Tween-20/TE) + 1/3 protease inhibitor cocktail, 1× with TE-NaCl (50 mM NaCl + TE) and 1× with IDTET (0.2% Tween-20, 10 mM Tris pH 8 and 0,1 mM EDTA). Samples were finally resuspended in TT buffer (10 mM Tris pH 8 + 0.05% Tween-20) before on-bead library preparation. For SALL1, SMAD4 and P300 ChIPs, 500,000 to 2 million nuclei were thawed on ice and resuspended in ice-cold RLNR1 buffer (20 mM Tris–HCl pH 7.5, 150 mM NaCl, 1 mM EDTA, 0.5 mM EGTA, 0.4% sodium deoxycholate, 1% NP-40, 0,1% SDS and 0.5 mM DTT) + 1x protease inhibitor cocktail/PMSF. Samples were sonicated in a 96-place microtube rack (Covaris cat. no. 500282) using a Covaris E220 for 20 cycles with the following setting: time 60 s, duty cycle 5.0, PIP 175, cycles 200, amplitude 0.0, velocity 0.0, dwell 0.0. Samples were recovered and spun down at maximum speed, 4 °C for 10 min. One percent of the lysate was kept as ChIP input. Ten microliters of Dynabead Protein A and 10 µl of Dynabead Protein G beads per sample were coupled to either 4 µg of SALL1 antibody (Abcam, ab41974), SMAD4 antibody (1 μg each of Cell Signaling Technology 46535 and 38454) or P300 antibody (1 µg each of EMD Millipore RW128 and Diagenode C15200211). Beads/antibody was added to each sample, which were then rotated overnight at 4 °C. The samples were washed with the following buffers: 3× RLNR1 + PIC/PMSF/DTT, 6× LWB-RCNR1 (10 mM Tris–HCl pH 7.5, 1 mM EDTA, 0.7% sodium deoxycholate, 1% NP-40 and 250 mM LiCl) + PIC/PMSF, 3× TET and 2× IDTET, and then resuspended in TT for on-bead library preparation. Libraries for ChIP and input samples were prepared with NEBNext Ultra II DNA library prep kit (NEB) reagents according to the manufacturer’s protocol on the beads suspended in 25 μL TT (10 mM Tris–HCl pH 7.5 and 0.05% Tween-20), with reagent volumes reduced by half. DNA was eluted and crosslinks reversed by adding 4 μl 10% SDS, 4.5 μl 5 M NaCl, 3 μl EDTA, 4 μl EGTA, 1 μl proteinase K (20 mg ml^−1^) and 16 μl water, incubating for 1 h at 55 °C, then 30 min to overnight at 65 °C. DNA was purified using 2 μl of SpeedBeads (GE Healthcare), diluted with 20% PEG8000, 1.5 M NaCl to final of 12% PEG, eluted with 25 μl TT. DNA contained in the eluate was then amplified for 12–14 cycles in 25 μl PCR reactions using NEBNext High-Fidelity 2× PCR Master Mix (NEB) and 0.5 mM each of primers Solexa 1GA and Solexa 1GB. Resulting libraries were size selected by gel excision to 200–500 bp, purified and single-end sequenced using a HiSeq 4000.

### Species conservation of enhancer and TF binding sites

The *Sall1* enhancer sequences were extracted from the mm10 genome using HOMER (v4.11.1) ‘homerTools extract’^54^ and then aligned to the NCBI nt database v5 using BLASTn^57^ by specifying *Homo sapiens* taxon ID 9606 and gap opening penalty at 5 and gap extension penalty at 2. We reported the top alignment of each sequence with E-value <0.01. For successfully aligned enhancers, we scanned through both mouse enhancers and human homologs with position weight matrices (PWMs) from the JASPAR database^58^ to compute PWM scores^59^. An array of PWM scores were computed for every sequence using MAGGIE (v1.1) ‘find_motif’ function^60^ and were used to identify motif matches based on a PWM score larger than four, meaning 16-fold more likely than random backgrounds to be bound by the corresponding TF. The motif matches at homologous positions were considered conserved between mouse and human.

### Data mapping

FASTQ files from sequencing experiments were mapped to mm10. RNA-seq files were mapped using STAR (v2.5.3a)^61^ with default parameters. ATAC-seq and Hi-C FASTQ files were trimmed before mapping with Bowtie 2 (v2.3.5.1); ATAC-seq files were trimmed to 30 bp, and Hi-C fastq files were trimmed at DpnII recognition sites (GATC). Following trimming, ATAC-seq and Hi-C FASTQ files were mapped using Bowtie 2 (ref. ^62^). After mapping, tag directories were created using the HOMER command makeTagDirectory.

### RNA-seq analysis

The gene expression raw counts were quantified by HOMER’s^54^ analyzeRepeats command with the option ‘-condenseGenes -count exons -noadj’. Differential gene expression was calculated using the HOMER command ‘getDiffExpression.pl’. Transcript per kilobase million (TPM) was quantified for all genes matching accession number to raw counts. DEGs were assessed with DESeq2 (ref. ^63^) at p-adj <0.05 and FC >2 where indicated. Genes with TPM <4 in all conditions were removed from analysis. Gene Ontology enrichment analyses were performed using Metascape (v3.5)^64^.

### IDR analysis of ChIP and ATAC peaks

ChIP–seq experiments were performed in replicates with corresponding input experiments. Peaks were called with HOMER for each tag directory with relaxed peak finding parameters ‘-L 0 -C 0 -fdr 0.9’. ATAC peaks were called with additional parameters ‘-minDist 200 -size 200’. IDR (Irreproducible Discovery Rate) (v2.0.4) was used to test for reproducibility between replicates^65^; only peaks with an IDR <0.05 were used for downstream analyses. For sample groups with more than two libraries, peak sets from all pairwise IDR comparisons were merged into a final set of peaks for further analysis.

### ATAC-seq and ChIP–seq analysis

To quantify the TF binding and chromatin accessibility between conditions, raw and normalized tag counts at merged IDR peaks identified by HOMER’s mergePeaks were identified using HOMER’s annotatePeaks with ‘-noadj’, ‘-size 500’ for TF ChIP–seq peaks and ‘-size 1000’ for ATAC peaks annotated with H3K27ac reads. DESeq2 was used to identify differentially bound TF binding distal sites or differential distal chromatin accessibility (p-adj <0.05 and FC >2 or <−2). SEs were defined using the HOMER ‘findPeaks -style super’ command.

### PLAC-seq analysis

H3K4me3 ChIP–seqs from purified ex vivo microglia were performed in duplicate with input controls. Alignment, quality control and peak calling were performed with the official ENCODE-ChIP-seq pipeline (v2.0.0) as previously described^18^. PLAC-seq fastq-files were processed with MAPS (v1.1.0)^66^ at 5,000 bp resolution as previously described^18^; the H3K4me3-ChIP–seq peak files from the ENCODE pipeline were used as a template.

### Motif analysis

To identify motifs enriched in peak regions over the background, HOMER’s motif analysis (findMotifsGenome.pl) including known default motifs and de novo motifs was used^54^. The background peaks used random genome sequences generated automatically by HOMER.

### Machine learning

The machine learning pipeline consisted of three primary stages: training data preparation, model training and model analysis. Training data preparation relied on HOMER^54^ for peak identifications and annotations and on Bedtools (v2.21.0)^67^ for sequence transformations. DeepSTARR^34^ was used for model training, and DeepLIFT^35^ was used for nucleotide contribution score analysis.

We used the convolutional neural network framework of DeepSTARR that was developed and tested for constructing (DNA sequence)-to-(enhancer activity) predictive models. The two fundamental variations in our modeling paradigm were in the categorical versus the regressive prediction form of the model output, *y* = F(**x**;**w**). The model output here, *y*, is a scalar variable corresponding to tag counts or sequence categories. The input, **x**, is the fixed-length DNA sequence, and **w** is the learned model parameter vector. The most informative results were obtained by training a regressive model to predict normalized ChIP–seq tag counts. We initially applied this approach to SALL1 ChIP–seq data. DNA segments were subselected from within ATAC peaks to construct the training dataset. To capture the full range of the data space, the training set included a large number of segments from both high and low ChIP–seq tag counts. The SALL1 model training set included approximately 200,000 DNA segments. Approximately 35% of the training set had SALL1 tag counts <2, and 65% had tag counts >60. The model fidelity was quantified using Pearson’s correlation coefficient, with SALL1 model yielding a Pearson’s correlation coefficient of 0.61. The SMAD4 model training set included approximately 185,000 DNA segments. Segments were subselected from within ATAC peaks. Approximately 55% of the training set had SMAD4 tag count <2, and 45% were segments with tag count >40.

SMAD4 model yielded a PPC of 0.41. Although lower than SALL1, the learning performance was sufficient to capture characteristics specific to SMAD4. Post model training, we derived nucleotide contribution scores using DeepLIFT. Nucleotide contribution scores were calculated on a select set of DNA segments.

### Motif mutation analysis

To integrate the genetic variation across mouse strains into motif analysis, we used MAGGIE, which is able to identify functional motifs out of the currently known motifs by testing for the association between motif mutations and the changes in specific epigenomic features^60^. The known motifs are obtained from the JASPAR database^58^. We applied this tool to strain-differential SALL1 peaks. Strain-differential SALL1 binding sites were defined by reproducible ChIP–seq peaks called in one strain but not in the other. ‘Positive sequences’ and ‘negative sequences’ were specified as sequences from the bound and unbound strains, respectively. The output *P* values with signs indicating directional associations were averaged for clusters of motifs grouped by a maximum correlation of motif score differences larger than 0.6. Only motif clusters with at least one member showing a corresponding gene expression higher than 2 TPM in microglia were considered as biologically relevant motifs.

### Statistical analyses

Gene expression differences and differential TF binding/H3K27ac signal were calculated with DESeq2 with Benjamini–Hochberg multiple testing correction. Genes and peaks were considered differential at FC >2 or <−2, p-adj <0.05. Significance of gene set overlap was calculated using the one-tailed Fisher exact test, *P* < 0.05.

### Reporting summary

Further information on research design is available in the Nature Portfolio Reporting Summary linked to this article.

## Online content

Any methods, additional references, Nature Portfolio reporting summaries, source data, extended data, supplementary information, acknowledgements, peer review information; details of author contributions and competing interests; and statements of data and code availability are available at 10.1038/s41590-023-01528-8.

## Supplementary information

Supplementary Information Supplementary Fig. 1 and additional methods.

Reporting Summary

Supplementary Table 1 Full list of microglia-specific genes, defined as being expressed ten-fold more highly than in other TRMs of interest.

## Data Availability

Previously reported data were downloaded from GEO and Array Express. Gosselin et al.^11^: GSE62826, Sajti et al.^24^: GSE137068, Sakai et al.^23^: GSE128662, Shemer et al.^25^: GSE122769, Buttgereit et al.^3^: E-MTAB-5077. Embryonic kidney H3K27ac from ENCODE Experiment ENCSR711SB was downloaded for visualization using the UCSC genome browser. Data generated by this study are accessible at GSE226092. Source data are provided with this paper.

## Source data

Source Data Statistical source data with tabs for each figure/extended data figure.

## Code availability

Code for the ENCODE PLAC-seq analysis pipeline is available at https://github.com/ENCODE-DCC/chip-seq-pipeline2.
