# Supplementary material for: SALL1 enforces microglia-specific DNA binding and function of SMADs to establish microglia identity
Source: Nat Immunol. 2023 Jun 15;24(7):1188–99. doi: 10.1038/s41590-023-01528-8 (PMC10307637; doi:10.1038/s41590-023-01528-8)
Supplement: Supplementary file 1 — Supplementary Fig. 1 and additional methods. [file 41590_2023_1528_MOESM1_ESM.pdf]

nature immunology

Resource

https://doi.org/10.1038/s41590-023-01528-8

SALL1 enforces microglia-specific DNA binding and function of SMADs to establish microglia identity

In the format provided by the authors and unedited

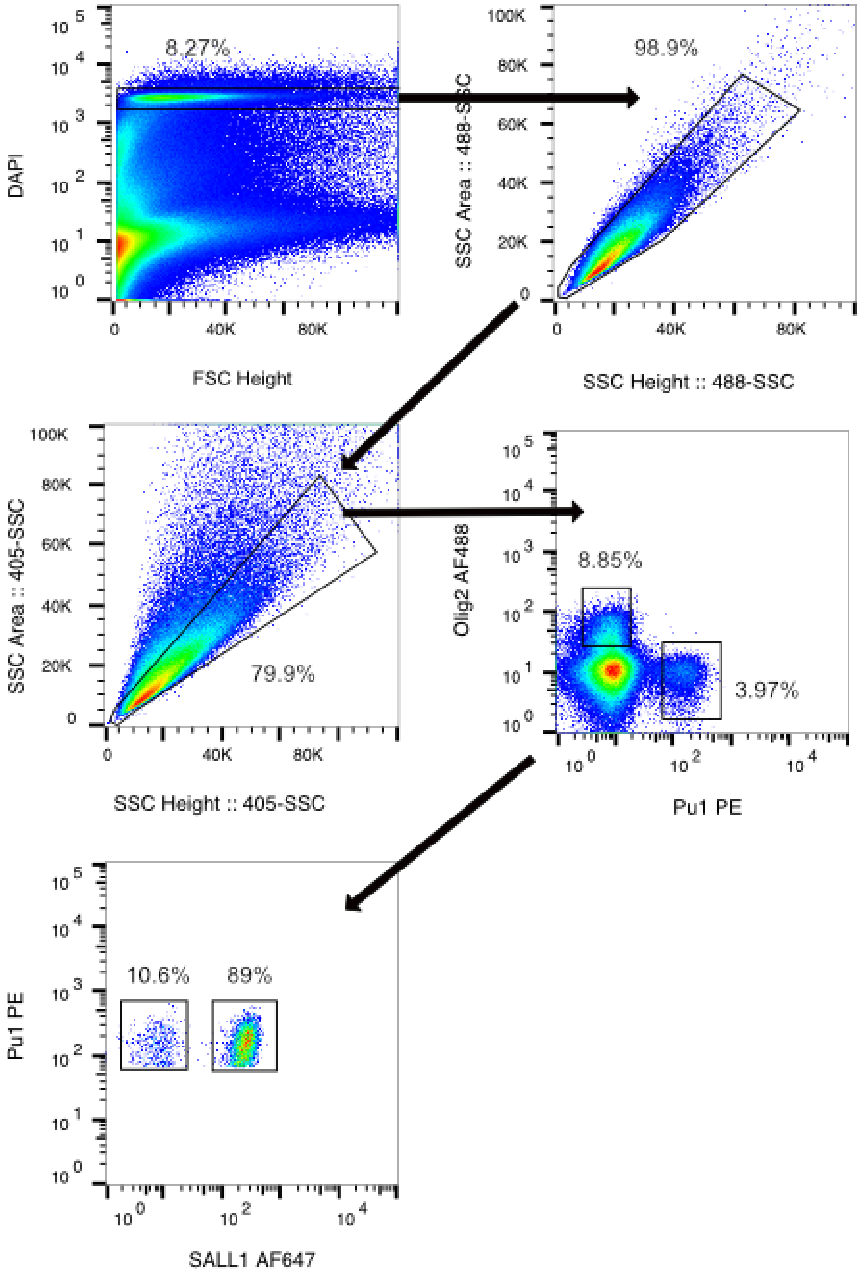

Supplementary Figure 1: FACS gating strategy for isolating SALL1+/PU1+ nuclei from mouse brain.

Additional Methods

smFISH for *Sall1*, *Cx3cr1* and *Csfr1*

8 weeks old female WT and *Sall1* EKO mice brains were dissected and freshly frozen in Neg-50 (epredia) for subsequent cryosection. 12 µm sections were cut on cryostat and collected on Poly-l-lysine coated cover glasses. Immediately fix samples on the cover glasses with 4% PFA for 10 min at 22°C. After 3 times wash with PBS, permeabilize samples with 80% Ethanol for 2 hrs at 22°C. Then wash samples with Pre-Wash solution (40% formamide, 2x SSC, 0.5%Triton) for 15 min. Hybridize samples with probes that targeting mRNAs of each gene (~20 µM in total concentration, about 48 to 99 different oligonucleotides were designed, and each gene's probe oligonucleotides were labeled with fluorophores. *Sall1* probes were labeled with Cy3, *Cx3cr1* probes were labeled with Cy5 and *Csfr1* probes were labeled with Alexa-750. Probe oligonucleotide pools were ordered from IDT) in hybridization solution (50% Formamide, 2x SSC and 10% Dextran sulfate). Incubate samples at 47°C overnight. Next day wash samples with Pre-Wash solution for 30 min at 22°C, followed with one wash of 2x SSC. Stain nucleus with 1 µM DAPI, and imaged on a custom-microscopy system consisting of a 500 frames per second, 6.5 micron x 6.5 micron pixel sized, 3200x3200 array camera, a CELESTA 1W laser system with 7 independent controlled laser light source for detection of DAPI, FITC, TRITC, Cy5, Cy7 AND spectrally similar fluorophores in combination with pentaband dichroic 10-10858, and with two Nikon 4x and 60x Objects.

Quantification of cell morphology and density

Deeply anesthetized mice were transcardially perfused at 22°C with 0.9% saline followed by freshly prepared 4% paraformaldehyde (PFA) in phosphate-buffered saline (PBS) and then post-fixed in 4% PFA at 4°C for 24 h followed by cryoprotection for 24h at 4°C in 20% sucrose. Brains were frozen in a plastic mold containing Tissue Freezing Medium (General Data Inc.), and quickly frozen in isopentane on dry ice. Briefly, 30µM coronal slices were collected and kept in 0.01M PBS at 4°C until use. Sectioned brains were scored according to their distance from Bregma (Franklin & Paxinos, 2007). Brain slices containing the prefrontal cortex (PFC) (AP: +2.8 to +1.8) were permeabilized in optimized detergent (e.g., 0.1% Triton-X-100) and blocked in normal horse serum (Thermo Fisher) for 1 hour at 22°C. Sections were then incubated with primary antibodies overnight at 4°C. Sections were then incubated with the corresponding secondary antibody for 2 h at 22°C. Finally, sections were mounted, dehydrated, and cover slipped with Immunoblot mounting medium (Thermo Fisher).Sections were imaged on a TCS SPE confocal microscope (Leica) (1024 × 1024 pixel, 16-bit depth, pixel size 0.63-micron, zoom 0.7). Images were acquired within brain regions using 40x-63x objectives. Z-stack images containing 6-10 microglia per ROI (40 µm depth, 1 µm steps, 40x magnification, n=15/brain region) were obtained. Raw files were used for further analysis using ImageJ (version 1.53j)[52]. Microglia were then segmented, and morphology was assessed in two-dimensional (2D) and three-dimensional (3D) space. For 2D analysis, maximum intensity projections of the XY planes were used. ImageJ's analysis for perimeter were measured in 2D for soma size and surface area was measured in 3D using the custom '3DShape' plugin as previously described[69]. Density was measured using maximum intensity projections of the XY plane and the ImageJ automated cell-counting plugin ITCN[70].

PLAC-seq library preparation

PLAC-seq libraries were prepared for *ex vivo* microglia as previously described with minor modifications[10,17,18]. Briefly, prior to FACS purification for microglia (1-2 million), harvested mouse brain cells were crosslinked for 15 min at 22°C with 1% formaldehyde and quenched for 5 min with 0.2M glycine, then centrifuged at 2,500xg for 5min, stained and sorted for microglia as previously described. Nuclei was isolated from microglia by incubation with 200 µL lysis buffer (10 mM Tris-HCl (pH 8.0), 10 mM NaCl, 0.2% IPEGAL CA-630) for 15 min on ice, then centrifuged at 2,500xg for 5 min, washed in 300 µL of lysis buffer, and centrifuged at 2,500xg for 5 min. Resulting pellet was resuspended in 50 µL 0.5% SDS and incubated at 62°C for 10 min. Following, 160 µL 1.56% Triton X-100 was added to the suspension and incubated for 15 mins at 37°C. Chromatin of the microglia was digested for 2 hours at 37°C with rotation upon addition of 25 µl of 10X NEBuffer 2 and 100 U MboI. Enzymes were inactivated by heating for 20 mins at 62°C. Biotinylation of fragmented ends were then achieved by adding 50 µL of a mix containing 0.3 mM biotin-14-dATP, 0.3 mM dCTP, 0.3 mM dTTP, 0.3 mM dGTP, and 0.8 U µl-1 Klenow. Mixture was incubated for 60 mins at 37°C with rotation. Ends were subsequently ligated by adding a 900 µL master mix containing 120 µL 10X T4 DNA ligase buffer (NEB), 100 µL 10% TritionX-100, 6 µL 20 mg ml-1 BSA, 10 µL 400 U µl- 1 T4 DNA Ligase (NEB, high concentration formula) and 664 µL H2O and incubated for 120 mins at 23°C with slow rotation. Resulting nuclei were pelleted for 5 mins at 4°C at 2,500xg. For the ChIP, nuclei were resuspended in RIPA Buffer (10 mM Tris (pH 8.0), 140 mM NaCl, 1 mM EDTA, 1% Triton X-100, 0.1% SDS, 0.1% sodium deoxycholate) with proteinase inhibitors and incubated on ice for 10 mins. Sonication was performed using a Covaris M220 instrument (Power 75W, duty factor 10%, cycle per bust 200, time 10 mins, temperature 7°C). Following, nuclei were spun down at 2500xg for 15 mins at 4°C. 5% of supernatant was taken as input DNA. Anti-H3K4me3 antibody-coated Dynabeads M-280 Sheep anti-Rabbit IgG (5 µg antibody per sample, Millipore, 04-745) was added to the remaining lysate, followed by rotation at 4°C overnight for immunoprecipitation. The sample was placed on a magnetic stand for 1 min and the beads were washed three times with RIPA buffer, two times with high-salt RIPA buffer (10 mM Tris pH 8.0, 300 mM NaCl, 1 mM EDTA, 1% Triton X-100, 0.1% SDS, 0.1% deoxycholate), one time with LiCl buffer (10 mM Tris (pH 8.0), 250 mM LiCl, 1 mM EDTA, 0.5% IGEPAL CA-630, 0.1% sodium deoxycholate) and two times with TE buffer (10 mM Tris (pH 8.0), 0.1 mM EDTA). Washed beads were treated with 10 µg RNase A in extraction buffer (10 mM Tris (pH 8.0), 350 mM NaCl, 0.1 mM EDTA, 1% SDS) for 1 hour at 37°C, and subsequently 20 µg proteinase K was added at 65°C for 2 hours. ChIP DNA was purified with Zymo DNA clean & concentrator. For Biotin pull down, 25 µL of 10 mg ml-1 Dynabeads My One T1 Streptavidin beads was washed with 400 µl of 1X Tween Wash Buffer (5 mM Tris-HCl (pH 7.5), 0.5 mM EDTA, 1 M NaCl, 0.05% Tween). Supernatant was then removed after separation on a magnet. Beads were resuspended in 2X Binding Buffer (10 mM Tris-HCl (pH 7.5), 1 mM EDTA, 2 M NaCl), added to the sample and incubated for 15 min at 22°C. Then beads were washed twice with 1X Tween Wash Buffer. Beads were heated on thermomixer for 2 mins at 55°C with mixing in between washings. Beads were then washed once with 1X NEB T4 DNA ligase buffer. Library prep was prepared using QIAseq Ultralow Input Library Kit (Qiagen, 180492). Concentration and cycle number for final

PCR was estimated through KAPA qPCR assay. Final PCR was directly amplified off the T1 beads according to the qPCR results. DNA was size selected with 0.5X and 1X SPRI Cleanup and eluted in 1X Tris Buffer and paired-end sequenced.

***In Situ* Hi-C Library Preparation**

In situ Hi-C was performed as described previously, with minor modifications[56]. Briefly, brain cells were crosslinked in 1% formaldehyde for 10 min at 22°C, quenched in 2.6M glycine, and washed twice with ice cold 0.5%BSA/PBS, followed by FACS sorting for microglia ($1x10^6$). Nuclei were then extracted from purified microglia through resuspension in 200 µL of wash buffer (50mM Tris HCl, pH 7.5, 10mM NaCl, 1mM EDTA, 0.5% SDS, 1x protease inhibitor cocktail (Sigma P8340)). Nuclei were incubated at 37°C for 60 minutes in PCR tubes in a thermocycler. Nuclei were spun down at 1000xg for 5 min at 22°C. Most of the supernatant was discarded, leaving 10µL of liquid with the nuclei. Samples were resuspended in DpnII buffer (25 µL 10% TrixonX-100, 25 µL 10x DpnII buffer (NEB), 188µL water) and rotated for 15 minutes at 37°C. Chromatin was then digested overnight with 2 µL (100U) DpnII (NEB) at 37°C, rotating end over end at 8 RPM. The next day, nuclei were spun down for 5 min, 1000xg. 225 µL of the supernatant was discarded, leaving ~25 µL of liquid remaining with the nuclei pellet. Overhangs were filled in with Biotin-14-dATP (ThermoFisher) by adding 75 µL of Klenow master mix (54.45 µL water, 7.5 µL NEBuffer 2 (NEB), 0.35 µL dCTP, 0.35 µL dTTP, 0.35 µL dGTP, 7.5 µL 0.4 mM Biotin-14-dATP, 2 µL 10% TritonX-100, 2.4 µL (12.5 U) Klenow Fragment (Enzymatics) and rotating end over end at room temperature for 40 minutes. Proximity ligation was performed by transferring the reaction into an Eppendorf tube and adding ligation master mix (322.75 µL water, 40 µL 10x T4 ligase buffer (Enzymatics), 36 µL 10% TritonX-100, 20% 1000x BSA, and HC T4 DNA ligase (Enzymatics). Samples were incubated at 16°C overnight rotating end over end. The following day, reactions were stopped by adding 20uL 0.5M EDTA plus 1 µL 10 µg/µL RNAse A at 42°C for 15 min. To reverse crosslinks and digest proteins, 31 µL 5M NaCl, 29 µL 10% SDS, and 5 µL 20mg/mL proteinase K were added to each sample. Samples were incubated at 55°C for 1 hour, then at 65°C overnight. The following day, DNA was extracted using 600 µL pH 8-buffered phenol/chloroform/isoamyl alcohol (Invitrogen), followed by 550 µL chloroform. DNA was then precipitated with 1.5 µL (15mg/mL) Glycoblue (Thermo) and 1400 µL 100% ethanol overnight at -20°C, pelleted for 20 minutes at 16000xg, 4°C and washed 2x with 1 mL 80% EtOH. The pellet was air-dried and dissolved in 131 µL TT buffer (0.05% Tween20/10 mM Tris pH 8). DNA was then sheared to ~300 bp average size in 130 µL TT on a Covaris E220 for 120 seconds, duty cycle 5 , PIP 175, and cycles per burst 200. Biotinylated DNA was incubated with 20uL DynaBeads MyOne Streptavidin T1 beads that had been washed 1x with B&W buffer (2x B&W: 10mM Tris HCl, pH 7.5, 1mM EDTA, 2M NaCl) and resuspended in 130 µL 2x B&W buffer with 0.2% Tween 20. The binding reaction was incubated for 45 minutes at room temperature, rotating end over end. The beads were washed 2x with 150 µL 1x B&W plus 0.1% TritonX-100, 1x with 180 µL TET (TE + 0.05% Tween-20) and resuspended in 30 µL ice cold NEBNext Ultra II end prep mix (1.5 µL NEBNext Ultra II EndPrep Enzyme, 3.5 µL EndPrep Buffer, 25 µL TT buffer) and incubated 20°C for 30 minutes followed by 65□C for 30 minutes. Beads were resuspended in ligation master mix (15 µL NebNext Ultra II ligation master mix, 0.5 µL ligation enhancer) and 1 µL of BIOO Nextflex DNA sequencing adapters were added. The

mixture was incubated at 20°C for 20 minutes and the reaction was stopped using 5 µL 0.5M EDTA. Following this, the beads were washed twice in 1x B&W with 0.1% TritonX-100, twice with TET, and resuspended in 20 µL TT buffer. Libraries were amplified by PCR for 10 cycles (98°C, 30 sec; [98°C, 10 sec; 63°C, 25 sec; 72°C, 30sec]; 72°C 5 minutes, 4°C hold) using 10 uL of the bead resuspension in a 50 µL reaction with NEBNext Ultra II Q5 mastermix (NEB), 0.5 µM each Solexa 1GA/1GB primers (Solexa 1GA: AATGATACGGCGACCACC GA, Solexa 1GB: CAAGCAGAAGA CGGCATACGA). Libraries were precipitated onto magnetic beads by adding 2 µL of Speedbeads, 40 µL 20% PEG/2.5M NaCl and incubating for 15 minutes at room temperature. The beads were washed 2x with 180 uL 80% EtOH and air dried. Samples were eluted by adding 20 µL TT buffer per sample. Libraries were sequenced to a depth of approximately 500 million paired end reads per experiment on Illumina NovaSeq.

Hi-C data Analysis and Visualization

Hi-C interaction matrices were generated using juicertools (v3.0)[71] and were visualized using juicebox (v.2.20.00)[72]. PC1 values for each sample were calculated using HOMER's runHiCpca.pl with -res 50000[73] and were visualized using the UCSC genome browser[74]. Differential PC1 compartments were determined using the command 'getHiCCorrDiff.pl". TADs and loops were called using HOMER's findTADsAndLoops.pl find with parameters -res 3000 and -window 15000. To compare TADs and loops between groups, TADs and loops were merged using merge2Dbed.pl -tad and -loop, respectively. Differential enrichment of these features was then calculated using Homer's getDiffExpression.pl.

Data Visualization

Heatmap of RNA expression or tags of ATAC-Seq peaks were generated by R package pheatmap (v1.0.12). Differentially expressed genes displayed in MA-plots using R, with dot size representing p-values. Data were further visualized using the HOMER command makeMultiWigHub.pl and the UCSC genome browser. GraphPad Prism (v9) was used to create barplots of TPMs in individual gene comparisons.

Additional Methods References

69 Sheets, K. G., Jun, B., Zhou, Y., Zhu, M., Petasis, N. A., Gordon, W. C. & Bazan, N. G. Microglial ramification and redistribution concomitant with the attenuation of choroidal neovascularization by neuroprotectin D1. *Mol Vis* **19**, 1747-1759 (2013).

70 Byun, J., Verardo, M. R., Sumengen, B., Lewis, G. P., Manjunath, B. S. & Fisher, S. K. Automated tool for the detection of cell nuclei in digital microscopic images: application to retinal images. *Mol Vis* **12**, 949-960 (2006).

71 Durand, N. C., Shamim, M. S., Machol, I., Rao, S. S. P., Huntley, M. H., Lander, E. S. & Aiden, E. L. Juicer Provides a One-Click System for Analyzing Loop-Resolution Hi-C Experiments. *Cell Systems* **3**, 95-98 (2016). https://doi.org:10.1016/j.cels.2016.07.002

72 Durand, N. C., Robinson, J. T., Shamim, M. S., Machol, I., Mesirov, J. P., Lander, E. S. & Aiden, E. L. Juicebox Provides a Visualization System for Hi-C Contact

Maps with Unlimited Zoom. *Cell Systems* **3**, 99-101 (2016). https://doi.org:10.1016/j.cels.2015.07.012
73 Lin, Y. C., Benner, C., Mansson, R., Heinz, S., Miyazaki, K., Miyazaki, M., Chandra, V., Bossen, C., Glass, C. K. & Murre, C. Global changes in the nuclear positioning of genes and intra- and interdomain genomic interactions that orchestrate B cell fate. *Nature Immunology* **13**, 1196-1204 (2012). https://doi.org:10.1038/ni.2432
74 Kent, W. J., Sugnet, C. W., Furey, T. S., Roskin, K. M., Pringle, T. H., Zahler, A. M. & Haussler, D. The human genome browser at UCSC. *Genome Research* **12**, 996-1006 (2002). https://doi.org:10.1101/gr.229102
