# Supplementary material for: SALL1 enforces microglia-specific DNA binding and function of SMADs to establish microglia identity
Source: Nat Immunol. 2023 Jun 15;24(7):1188–99. doi: 10.1038/s41590-023-01528-8 (PMC10307637; doi:10.1038/s41590-023-01528-8)
Supplement: Supplementary file 2 — Reporting Summary [file 41590_2023_1528_MOESM2_ESM.pdf]

nature portfolio

Corresponding author(s): Christopher K. Glass

Last updated by author(s): Apr 28, 2023

Reporting Summary

Nature Portfolio wishes to improve the reproducibility of the work that we publish. This form provides structure for consistency and transparency in reporting. For further information on Nature Portfolio policies, see our Editorial Policies and the Editorial Policy Checklist.

Statistics

For all statistical analyses, confirm that the following items are present in the figure legend, table legend, main text, or Methods section.

n/a	Confirmed	
☐	☒	The exact sample size (*n*) for each experimental group/condition, given as a discrete number and unit of measurement
☐	☒	A statement on whether measurements were taken from distinct samples or whether the same sample was measured repeatedly
☐	☒	The statistical test(s) used AND whether they are one- or two-sided<br>*Only common tests should be described solely by name; describe more complex techniques in the Methods section.*
☐	☒	A description of all covariates tested
☐	☒	A description of any assumptions or corrections, such as tests of normality and adjustment for multiple comparisons
☐	☒	A full description of the statistical parameters including central tendency (e.g. means) or other basic estimates (e.g. regression coefficient) AND variation (e.g. standard deviation) or associated estimates of uncertainty (e.g. confidence intervals)
☐	☒	For null hypothesis testing, the test statistic (e.g. *F*, *t*, *r*) with confidence intervals, effect sizes, degrees of freedom and *P* value noted<br>*Give P values as exact values whenever suitable.*
☒	☐	For Bayesian analysis, information on the choice of priors and Markov chain Monte Carlo settings
☐	☒	For hierarchical and complex designs, identification of the appropriate level for tests and full reporting of outcomes
☐	☒	Estimates of effect sizes (e.g. Cohen's *d*, Pearson's *r*), indicating how they were calculated

Our web collection on statistics for biologists contains articles on many of the points above.

Software and code

Policy information about availability of computer code

Data collection	Flow cytometry data was collected on Sony MA900 or MoFlo Astrios EQ cell sorter. Imaging data was collected on a Nikon Sterling Spinning Disk Confocal Microscope with 60x object, TCS SPE confocal microscope (Leica) or a custom-microscopy system consisting of a 500 frames per second, 6.5 micron x 6.5 micron pixel sized, 3200x3200 array camera, a CELESTA 1W laser system with 7 independent controlled laser light source for detection of DAPI, FITC, TRITC, Cy5, Cy7 AND spectrally similar fluorophores in combination with pentaband dichroic 10-10858, and with two Nikon 4x and 60x Objects.
Data analysis	Data preprocessing<br>FASTQ files from sequencing experiments were mapped to mm10. RNA-seq files were mapped using STAR (2.5.3a) with default parameters. ATAC-seq and Hi-C FASTQ files were trimmed prior to mapping with Bowtie 2 (2.3.5.1); ATAC-seq files were trimmed to 30 bp and Hi-C fastq files were trimmed at DpnII recognition sites (GATC). Following trimming, ATAC-seq and Hi-C FASTQ files were mapped using Bowtie 2 (2.3.5.1). After mapping, tag directories were created using the HOMER (v4.11.1) command makeTagDirectory.<br><br>RNA-seq Analysis<br>The gene expression raw counts were quantified by HOMER's (v4.11.1) analyzeRepeats command with the option "-condenseGenes -count exons -noadj". Differential gene expression was calculated using the HOMER command "getDiffExpression.pl". TPM (transcript per kilobase million) were quantified for all genes matching accession number to raw counts. Differentially expressed genes were assessed with DESeq2 at p-adj (adjusted pvalue) < 0.05 and FC (fold change) > 2 where indicated. Genes with TPM < 4 in all conditions were removed from analysis. Gene ontology enrichment analyses were performed using Metascape (v3.5).<br><br>IDR analysis of ChIP and ATAC peaks<br>ChIP-seq experiments were performed in replicates with corresponding input experiments. Peaks were called with HOMER (v4.11.1) for each tag directory with relaxed peak finding parameters "-L 0 -C 0 -fdr 0.9". ATAC peaks were called with additional parameters "-minDist 200 -size 200". IDR (v2.0.4) was used to test for reproducibility between replicates, only peaks with an IDR < 0.05 were used for downstream analyses. For sample groups with > 2 libraries, peak sets from all pairwise IDR comparisons were merged into a final set of peaks for further analysis.

ATAC-seq and ChIP-seq analysis
To quantify the TF binding and chromatin accessibility between conditions, raw and normalized tag counts at merged IDR peaks identified by HOMER's (v4.11.1) mergePeaks were identified using HOMER's annotatePeaks with "-noadj," "-size 500" for TF ChIP-seq peaks and "-size 1000" for ATAC peaks annotated with H3K27ac reads. DESeq2 was used to identify differentially bound TF binding distal sites or differential distal chromatin accessibility (p-adj. < 0.05 and FC >2 or <-2). Super-enhancers were defined using the HOMER 'findPeaks -style super' command.

Motif Analysis
To identify motifs enriched in peak regions over the background, HOMER's motif analysis (findMotifsGenome.pl) including known default motifs and de novo motifs was used. The background peaks used random genome sequences generated automatically by HOMER (v4.11.1).

Conservation of enhancer sequences and TF binding sites between mouse and human
The Sall1 enhancer sequences were extracted from the mm10 genome using HOMER (v4.11.1) "homerTools extract" and then aligned to the NCBI nt database (v5) using BLASTn by specifying homo sapiens taxon ID 9606 and gap opening penalty at 5 and gap extension penalty at 2. We reported the top alignment of each sequence with E-value < 0.01. For successfully aligned enhancers, we scanned through both mouse enhancers and human homologs with position weight matrices (PWMs) from the JASPAR database to compute PWM scores. An array of PWM scores were computed for every sequence using MAGGIE (v1.1) "find_motif" function and were used to identify motif matches based on a PWM score larger than four, meaning 16-fold more likely than random backgrounds to be bound by the corresponding TF. The motif matches at homologous positions were considered conserved between mouse and human.

Hi-C data Analysis and Visualization
Hi-C interaction matrices were generated using juicertools (v3.0) and were visualized using juicebox (v.2.20.00). PC1 values for each sample were calculated using HOMER's runHiCpca.pl with -res 50000 and were visualized using the UCSC genome browser. Differential PC1 compartments were determined using the command 'getHiCCorrDiff.pl". TADs and loops were called using HOMER's findTADsAndLoops.pl find with parameters -res 3000 and -window 15000. To compare TADs and loops between groups, TADs and loops were merged using merge2Dbed.pl -tad and -loop, respectively. Differential enrichment of these features was then calculated using Homer's getDiffExpression.pl.

PLAC-seq Analysis
H3K4me3 ChIP-seqs from purified ex-vivo microglia were performed in duplicate with input controls. Alignment, QC and peak calling were performed with the official ENCODE-ChIP-seq pipeline (v2.0.0). PLAC-seq fastq-files were processed with MAPS (v1.1.0) at 5000-bp resolution as previously described; the H3K4me3-ChIP-seq peak files from the ENCODE pipeline were used as a template. Code for the ENCODE PLAC-seq analysis pipeline is available here: (https://github.com/ENCODE-DCC/chip-seq-pipeline2).

Statistical Analyses
Gene expression differences and differential TF binding/H3K27ac signal was calculated with DESeq2 (v1.12.4) with Benjamini-Hochberg multiple testing correction. Genes and peaks were considered differential at FC >2 or <-2, p.adj < 0.05. Significance of gene set overlap was calculated using the Fisher exact test, p.value < 0.05.

Motif mutation analysis
To integrate the genetic variation across mouse strains into motif analysis, we used MAGGIE (v1.1), which is able to identify functional motifs out of the currently known motifs by testing for the association between motif mutations and the changes in specific epigenomic features21. The known motifs are obtained from the JASPAR database19. We applied this tool to strain-differential SALL1 peaks. Strain-differential SALL1 binding sites were defined by reproducible ChIP-seq peaks called in one strain but not in the other. "Positive sequences" and "negative sequences" were specified as sequences from the bound and unbound strains, respectively. The output p-values with signs indicating directional associations were averaged for clusters of motifs grouped by a maximum correlation of motif score differences larger than 0.6. Only motif clusters with at least one member showing a corresponding gene expression larger than 2 TPM in microglia were considered as biologically relevant motifs.

Machine learning
The machine learning pipeline consisted of three primary stages: training data preparation, model training, and model analysis. Training data preparation relied on HOMER for peak identifications and annotations and on Bedtools (v2.21.0) for sequence transformations. DeepSTARR was used for model training, and DeepLIFT was used for nucleotide contribution score analysis. No version histories indicated for DeepSTARR and DeepLIFT.

We used the convolutional neural network (CNN) framework of DeepSTARR that was developed and tested for constructing (DNA sequence)-to-(enhancer activity) predictive models. The two fundamental variations in our modeling paradigm were in the categorical vs. the regressive prediction form of the model output, y=F(x;w). The model output here, y, is a scalar variable corresponding to tag counts or sequence categories. The input, x, is the fixed length DNA sequence, and w is the learned model parameter vector. The most informative results were obtained by training a regressive model to predict normalized ChIP-seq tag counts. We initially applied this approach to SALL1 ChIP-seq data. DNA segments were sub-selected from within ATAC peaks to construct the training data set. To capture the full range of the data space, the training set included a large number of segments from both high and low ChIP-seq tag counts. The SALL1 model training set included approximately 200K DNA segments. Approximately 35% of the training set had SALL1 tag counts < 2, and 65% had tag counts > 60. The model fidelity was quantified using Pearson's correlation coefficient (PCC), with SALL1 model yielding a PCC of 0.61. The SMAD4 model training set included approximately 185K DNA segments. Segments were sub-selected from within ATAC peaks. Approximately 55% of the training set had SMAD4 tag count < 2, and 45% were segments with tag count > 40. SMAD4 model yielded a PPC of 0.41. Although lower than SALL1, the learning performance was sufficient to capture characteristics specific to SMAD4. Post model training, we derived nucleotide contribution scores using DeepLIFT. Nucleotide contribution scores calculated on a select set of DNA segments.

For manuscripts utilizing custom algorithms or software that are central to the research but not yet described in published literature, software must be made available to editors and reviewers. We strongly encourage code deposition in a community repository (e.g. GitHub). See the Nature Portfolio guidelines for submitting code & software for further information.

Data

Policy information about availability of data

All manuscripts must include a data availability statement. This statement should provide the following information, where applicable:

- Accession codes, unique identifiers, or web links for publicly available datasets
- A description of any restrictions on data availability
- For clinical datasets or third party data, please ensure that the statement adheres to our policy

Data Availability
Data generated by this study is accessible at GSE226092. Previously reported data were downloaded from NCBI GEO. Gosselin et al.: GSE62826, Sajti et al.: GSE137068, Sakai et al.: GSE128662, Shemer et al.: GSE122769, Buttgereit et al.: E-MTAB-5077.

Field-specific reporting

Please select the one below that is the best fit for your research. If you are not sure, read the appropriate sections before making your selection.

☒ Life sciences ☐ Behavioural & social sciences ☐ Ecological, evolutionary & environmental sciences

For a reference copy of the document with all sections, see nature.com/documents/nr-reporting-summary-flat.pdf

Life sciences study design

All studies must disclose on these points even when the disclosure is negative.

Sample size	For all experiments, no statistical methods were used to pre-determine sample size but our sample sizes are similar to those reported in previous publications (Sakai et al. 2019, Gosselin et al., 2014).
Data exclusions	The reported data sets are from sequential samples for which cell viability and sequencing libraries met technical quality standards. No other criteria were used to include or exclude samples.
Replication	For RNA-seq studies, 2-4 biologically independent samples per group were used. For ATAC-seq, 5 biologically independent samples were used. For H3K27ac ChIP-seq, 2 biologically independent samples per group were used. For SALL1 and SMAD4 ChIP, 2 biologically independent samples per group were used. For Hi-C, 2 biologically independent samples per group were used. For PLAC-seq, two biologically independent WT samples were used. All assays were successfully replicated 2-3 times; quantification and statistics are run on combined replicate experiments.
Randomization	No randomization was performed. PCA analysis was used to determine potential confounders.
Blinding	We did not perform blinded studies as all mice received identical treatments.

Reporting for specific materials, systems and methods

We require information from authors about some types of materials, experimental systems and methods used in many studies. Here, indicate whether each material, system or method listed is relevant to your study. If you are not sure if a list item applies to your research, read the appropriate section before selecting a response.

Materials & experimental systems

n/a	Involved in the study	
☐	☒	Antibodies
☒	☐	Eukaryotic cell lines
☒	☐	Palaeontology and archaeology
☐	☒	Animals and other organisms
☒	☐	Human research participants
☒	☐	Clinical data
☒	☐	Dual use research of concern

Methods

n/a	Involved in the study	
☐	☒	ChIP-seq
☐	☒	Flow cytometry
☒	☐	MRI-based neuroimaging

Antibodies

Antibodies used	anti-CD16/32 blocking antibody (Biolegend 101319) 1:100<br>CD11b-APC, clone M1/70, (Biolegend 101212) 1:100<br>CD45-Alexa Fluor 488, clone 30-F11, (Biolegend 103122) 1:100<br>CX3CR1-PE, clone SA011F11, (Biolegend 149006) 1:100<br>Rabbit polyclonal Anti-Iba1 (FujiFilm 019-19741) 1:500

	Rat anti-mouse SALL1, clone NRNSTNX, (Thermo Fisher, 14-9729-82) 1:300<br>Donkey anti-Rat polyclonal DyLight 550 (Invitrogen SA5-10027) 1:500<br>Donkey anti-Rabbit polyclonal AF488 (Invitrogen R37118) 1:500<br>Goat anti-IBA1, polyclonal, (Abcam ab5076) 1:200<br>Donkey anti-Rat IgG Alexa Fluor 488, polyclonal, (Jackson ImmunoResearch, 712-545-150) 1:500<br>Donkey anti-Goat IgG Alexa Fluor 488, polyclonal, (Jackson ImmunoResearch, 705-545-147) 1:500<br>PU.1-PE, clone 9G7 (Cell Signaling 81886S) 1:100<br>OLIG2-AF488, clone EPR2673, (Abcam 225099) 1:2500<br>SALL1 AF647, clone NRNSTNX,(ThermoFisher 51-9279-82) 1:100<br>NEUN-AF488, clone A60, (Millipore MAB377X) 1:500<br>H3K27ac, clone MABI 0309, (Active Motif 39685) 1ug<br>SALL1, clone K9814, (Abcam ab41974) 4ug<br>SMAD4, clone D3R4N, (Cell Signaling technology 46535) 1ug<br>SMAD4, clone D3M6U, (Cell Signaling technology 38454) 1ug<br>P300, clone RW128, (EMD Millipore RW128) 1ug<br>P300, unknown clone, (Diagenode C15200211) 1ug
Validation	anti-CD16/32 blocking antibody (Biolegend 101319) - validated by manufacturer<br>CD11b-APC, clone M1/70, (Biolegend 101212) - validated by manufacturer<br>CD45-Alexa Fluor 488, clone 30-F11, (Biolegend 103122) - validated by manufacturer<br>CX3CR1-PE, clone SA011F11, (Biolegend 149006) - validated by manufacturer<br>Rabbit polyclonal Anti-Iba1 (FujiFilm 019-19741) - validated by manufacturer<br>Rat anti-mouse SALL1, clone NRNSTNX, (Thermo Fisher, 14-9729-82) - validated in house<br>Donkey anti-Rat polyclonal DyLight 550 (Invitrogen SA5-10027) - validated by manufacturer<br>Donkey anti-Rabbit polyclonal AF488 (Invitrogen R37118) - validated by manufacturer<br>Goat anti-IBA1, polyclonal, (Abcam ab5076) - validated by manufacturer<br>Donkey anti-Rat IgG Alexa Fluor 488, polyclonal, (Jackson ImmunoResearch, 712-545-150) - validated by manufacturer<br>Donkey anti-Goat IgG Alexa Fluor 488, polyclonal, (Jackson ImmunoResearch, 705-545-147) - validated by manufacturer<br>PU.1-PE, clone 9G7 (Cell Signaling 81886S) - validated by manufacturer<br>OLIG2-AF488, clone EPR2673, (Abcam 225099) - validated by manufacturer<br>SALL1 AF647, clone NRNSTNX,(ThermoFisher 51-9279-82) - validated in house<br>NEUN-AF488, clone A60, (Millipore MAB377X) 1:500 - validated by manufacturer<br>H3K27ac, clone MABI 0309, (Active Motif 39685) - validated by manufacturer<br>SALL1, clone K9814, (Abcam ab41974) 4ug - validated in house<br>SMAD4, clone D3R4N, (Cell Signaling technology 46535) - validated by manufacturer<br>SMAD4, clone D3M6U, (Cell Signaling technology 38454) - validated by manufacturer<br>P300, clone RW128, (EMD Millipore RW128) - validated by manufacturer<br>P300, unknown clone, (Diagenode C15200211) - validated by manufacturer

Animals and other organisms

Policy information about studies involving animals; ARRIVE guidelines recommended for reporting animal research

Laboratory animals	The following mice were used in this study: C57BL/6J (The Jackson Laboratory, Stock No. 00064), SPRET/EiJ (The Jackson Laboratory, Stock No. 001146), PWK/PhJ (The Jackson Laboratory, Stock No. 003715), Sall1 EKO (generated by Glass lab and transgenic core facility, University of California, San Diego), Cx3cr1CreER (The Jackson Laboratory, Stock No. 020940), and Smad4fl/fl (The Jackson Laboratory, Stock No. 017462). For experiments with C57BL/6J and Sall1 EKO, male mice were used between 8-12 weeks of age. Experiments for targeted, inducible deletion of Smad4 were performed on male mice at P0 and mice were harvested at 2 weeks of age.
Wild animals	No wild animals were used in this study.
Field-collected samples	No field-collected samples were used in this study.
Ethics oversight	All animal procedures were approved by the University of California San Diego Institutional Animal Care and Use Committee in accordance with University of California San Diego research guidelines for the care and use of laboratory animals.

Note that full information on the approval of the study protocol must also be provided in the manuscript.

ChIP-seq

Data deposition

☒ Confirm that both raw and final processed data have been deposited in a public database such as GEO.

☒ Confirm that you have deposited or provided access to graph files (e.g. BED files) for the called peaks.

Data access links<br>*May remain private before publication.*	Data generated by this study is accessible at GSE226092 (GEO).
Files in database submission	Raw files<br>WT_RNAseq_Rep1.fastq.gz

WT_RNAseq_Rep2.fastq.gz
WT_RNAseq_Rep3.fastq.gz
HetEKO_RNAseq_Rep1.fastq.gz
HetEKO_RNAseq_Rep2.fastq.gz
HetEKO_RNAseq_Rep3.fastq.gz
EKO_RNAseq_Rep1.fastq.gz
EKO_RNAseq_Rep2.fastq.gz
EKO_RNAseq_Rep3.fastq.gz
Smad4WT_RNAseq_Rep1.fastq.gz
Smad4WT_RNAseq_Rep2.fastq.gz
Smad4WT_RNAseq_Rep3.fastq.gz
Smad4WT_RNAseq_Rep4.fastq.gz
Smad4cKO_RNAseq_Rep1.fastq.gz
Smad4cKO_RNAseq_Rep2.fastq.gz
Smad4cKO_RNAseq_Rep3.fastq.gz
WT_ATAC_Rep1.fastq.gz
WT_ATAC_Rep2.fastq.gz
WT_ATAC_Rep3.fastq.gz
WT_ATAC_Rep4.fastq.gz
WT_ATAC_Rep5.fastq.gz
EKO_ATAC_Rep1.fastq.gz
EKO_ATAC_Rep2.fastq.gz
EKO_ATAC_Rep3.fastq.gz
EKO_ATAC_Rep4.fastq.gz
EKO_ATAC_Rep5.fastq.gz
WT_H3K27ac_Rep1.fastq.gz
WT_H3K27ac_Rep2.fastq.gz
WT_H3K27ac_Input_Rep1.fastq.gz
WT_H3K27ac_Input_Rep2.fastq.gz
EKO_H3K27ac_Rep1.fastq.gz
EKO_H3K27ac_Rep2.fastq.gz
EKO_H3K27ac_Input_Rep1.fastq.gz
EKO_H3K27ac_Input_Rep2.fastq.gz
WT_NeuN_H3K27ac_rep1.fastq.gz
WT_NeuN_H3K27ac_Rep2.fastq.gz
WT_NeuN_H3K27ac_Input_Rep1_R1.fastq.gz
WT_NeuN_H3K27ac_Input_Rep1_R2.fastq.gz
WT_NeuN_H3K27ac_Input_Rep2.fastq.gz
WT_Olig2_H3K27ac_rep1.fastq.gz
WT_Olig2_H3K27ac_Rep2.fastq.gz
WT_Olig2_H3K27ac_Input_Rep1_R1.fastq.gz
WT_Olig2_H3K27ac_Input_Rep1_R2.fastq.gz
WT_Olig2_H3K27ac_Input_Rep2.fastq.gz
WT_Pu1_H3K27ac_rep1.fastq.gz
WT_Pu1_H3K27ac_rep2.fastq.gz
WT_Pu1_H3K27ac_Input_Rep1_R1.fastq.gz
WT_Pu1_H3K27ac_Input_Rep1_R2.fastq.gz
WT_Pu1_H3K27ac_Input_Rep2.fastq.gz
EKO_NeuN_H3K27ac_Rep1.fastq.gz
EKO_NeuN_H3K27ac_Rep2.fastq.gz
EKO_NeuN_H3K27ac_Input_Rep1_R1.fastq.gz
EKO_NeuN_H3K27ac_Input_Rep1_R2.fastq.gz
EKO_NeuN_H3K27ac_Input_Rep2_R1.fastq.gz
EKO_NeuN_H3K27ac_Input_Rep2_R2.fastq.gz
EKO_Olig2_H3K27ac_Rep1.fastq.gz
EKO_Olig2_H3K27ac_Rep2.fastq.gz
EKO_Olig2_H3K27ac_Input_Rep1_R1.fastq.gz
EKO_Olig2_H3K27ac_Input_Rep1_R2.fastq.gz
EKO_Olig2_H3K27ac_Input_Rep2_R1.fastq.gz
EKO_Olig2_H3K27ac_Input_Rep2_R2.fastq.gz
EKO_PU1_H3K27ac_rep1.fastq.gz
EKO_PU1_H3K27ac_rep2.fastq.gz
EKO_Pu1_H3K27ac_Input_Rep1_R1.fastq.gz
EKO_Pu1_H3K27ac_Input_Rep1_R2.fastq.gz
EKO_Pu1_H3K27ac_Input_Rep2_R1.fastq.gz
EKO_Pu1_H3K27ac_Input_Rep2_R2.fastq.gz
WT_P300_Rep1.fastq.gz
WT_P300_Rep2.fastq.gz
WT_P300_Rep3.fastq.gz
WT_P300_Input_Rep1.fastq.gz
WT_P300_Input_Rep2.fastq.gz
WT_P300_Input_Rep3.fastq.gz
WT_SALL1_Rep1.fastq.gz
WT_SALL1_Rep2.fastq.gz
WT_SALL1_SMAD4_Input_Rep1.fastq.gz
WT_SALL1_Input_Rep2.fastq.gz

EKO_SALL1_Rep1.fastq.gz
EKO_SALL1_Rep2.fastq.gz
EKO_SALL1_Input_Rep1.fastq.gz
EKO_SALL1_Input_Rep2.fastq.gz
PWK_SALL1_Rep1.fastq.gz
PWK_SALL1_Rep2.fastq.gz
PWK_SALL1_Input_Rep1.fastq.gz
PWK_SALL1_Input_Rep2.fastq.gz
Spret_SALL1_Rep1.fastq.gz
Spret_SALL1_Rep2.fastq.gz
Spret_SALL1_Input_Rep1.fastq.gz
Spret_SALL1_Input_Rep2.fastq.gz
WT_SMAD4_Rep1.fastq.gz
WT_SMAD4_Rep2.fastq.gz
WT_SMAD4_Input_Rep2.fastq.gz
EKO_SMAD4_Rep1.fastq.gz
EKO_SMAD4_Rep2.fastq.gz
EKO_SMAD4_Input_Rep1.fastq.gz
EKO_SMAD4_Input_Rep2.fastq.gz
WT_HiC_Rep1_R1.fastq.gz
WT_HiC_Rep1_R2.fastq.gz
WT_HiC_Rep2_R1.fastq.gz
WT_HiC_Rep2_R2.fastq.gz
EKO_HiC_Rep1_R1.fastq.gz
EKO_HiC_Rep1_R2.fastq.gz
EKO_HiC_Rep2_R1.fastq.gz
EKO_HiC_Rep2_R2.fastq.gz
H3K4me3_Rep1.fastq.gz
H3K4me3_Rep2.fastq.gz
H3K4me3_Input_Rep1.fastq.gz
H3K4me3_Input_Rep2.fastq.gz
WT_PLAC_Rep1_R1.fastq.gz
WT_PLAC_Rep1_R2.fastq.gz
WT_PLAC_Rep2_R1.fastq.gz
WT_PLAC_Rep2_R2.fastq.gz

Processed Files (includes TPM, raw counts, peak files, IDR peak files)
SALL1SMAD4_RNAseq_RAW.txt
SALL1SMAD4_RNAseq_TPM.txt
Microglia_H3K27acATAC_Norm.txt
WT_Pu1_H3K27ac_rep1
WT_Pu1_H3K27ac_rep2
WT_NeuN_H3K27ac_rep1
WT_NeuN_H3K27ac_Rep2
WT_Olig2_H3K27ac_rep1
WT_Olig2_H3K27ac_Rep2
EKO_Pu1_H3K27ac_rep1
EKO_Pu1_H3K27ac_rep2
EKO_NeuN_H3K27ac_Rep1
EKO_NeuN_H3K27ac_Rep2
EKO_Olig2_H3K27ac_Rep1
EKO_Olig2_H3K27ac_Rep2
WT_SALL1_idr_peaks.txt
EKO_SALL1_idr_peaks.txt
PWK_SALL1_idr_peaks.txt
Spret_SALL1_idr_peaks.txt
WT_SMAD4_idr_peaks.txt
EKO_SMAD4_idr_peaks.txt
WT_P300_idr_peak.peak
H3K4me3_Rep1
H3K4me3_Rep2
WT_Combined.hic
KO_Combined.hic
Microglia.5k.2.peaks.annotated.bedpe
SALL1_WT.idr
SALL1_EKO.idr

Genome browser session
(e.g. UCSC)

https://genome.ucsc.edu/cgi-bin/hgTracks?db=mm10&lastVirtModeType=default&lastVirtModeExtraState=&virtModeType=default&virtMode=0&nonVirtPosition=&position=chr8%3A88953844%2D89593622&hgsid=1325471843_xOYsbz6WnIdZavQnSP6fu0A7DUXy

Methodology

Replicates	Each ChIP experiment contains 2 biological replicates per group, with the exception of P300, WT PU1 H3K27ac, WT Olig2 H3K27ac, and WT NeuN H3K27aac (1 biological replicate per group)
Sequencing depth	Samples were sequenced using Illumina HiSeq4000 or NOVA-seq single/paired end sequencer. The total read numbers for each sample range between 11M-60M.
Antibodies	H3K27ac (Active Motif 39685).<br>SALL1(Abcam, ab41974)<br>SMAD4 (Cell Signaling technology 46535)<br>SMAD4 (Cell Signaling technology 38454)<br>P300 (EMD Millipore RW128)<br>P300 (Diagenode C15200211)
Peak calling parameters	Fastq reads were mapped to hg38 genome build with default parameters. Aligned reads were saved in sam files and subsequently converted to tag directories with HOMER. Peaks were called using HOMER findPeaks function with matched input files and the following parameters "L 0 -C 0 -fdr 0.9".
Data quality	ChIP-seq with replicates were filtered using Irreproducible Discovery Rate (IDR., Peaks with IDR>=0.05 were filtered).
Software	HOMER

Flow Cytometry

Plots

Confirm that:

☒ The axis labels state the marker and fluorochrome used (e.g. CD4-FITC).

☒ The axis scales are clearly visible. Include numbers along axes only for bottom left plot of group (a 'group' is an analysis of identical markers).

☒ All plots are contour plots with outliers or pseudocolor plots.

☒ A numerical value for number of cells or percentage (with statistics) is provided.

Methodology

Sample preparation	Mouse brains were homogenized as previously described by gentle mechanical dissociation. Cells were then incubated in staining buffer on ice with anti-CD16/32 blocking antibody (BioLegend 101319) for 15 minutes, and then with anti-mouse anti-CD11b-APC (BioLegend 101212), anti-CD45-Alexa488 (BioLegend 103122), and anti-CX3CR1-PE (BioLegend 149006) for 25 minutes. Cell preparations for H3K27ac ChIP-seq, PLAC-seq, and Hi-C were fixed with 1% formaldehyde for 10 minutes and quenched with 0.125M glycine for 5 minutes after staining, and subsequently washed three times. Cells were washed once and filtered through a 40 uM cell strainer. Sorting was performed on a Sony MA900 or MoFlo Astrios EQ cell sorter. Microglia were defined as events that were DAPI negative, singlets, and CD11b+CD45lowCX3CR1+. Isolated microglia were then processed according to protocols for RNA-seq, ATAC-seq and ChIP-seq, Hi-C, and PLAC-seq.<br><br>Brain nuclei were isolated as previously described with initial homogenization performed with either 1% formaldehyde in Dulbecco's phosphate buffered saline or 2mM DSG (Proteochem) in Dulbecco's phosphate buffered saline. Nuclei were stained overnight with PU.1-PE (Cell Signaling 81886S), OLIG2-AF488 (Abcam 225099) or SALL1 AF647 (Thermo, clone NRNSTNX 51-9279-82) or NEUN-AF488 (Millipore MAB 377X). Nuclei were washed the following day with 4 mL FACs buffer, passed through a 40 uM strainer, and stained with 0.5 ug/mL DAPI. Nuclei for each cell type were sorted with a Beckman Coulter MoFlo Astrio EQ cell sorter and pelleted at 1600xg for 5 minutes at 4°C in FACs buffer. Nuclei pellets were snap frozen and stored at -80°C prior to library preparation.
Instrument	Beckman Coulter MoFlo Astrios EQ cell sorter, SONY MA900 cell sorter
Software	FlowJoV10.4.1
Cell population abundance	Whole, live microglia constituted 8-16% of the total events sorted. SALL1+PU1+ nuclei composed approximately 4.5-5% of the total events sorted, while PU1+SALL1negative nuclei composed 0.4-0.5% of total events sorted.
Gating strategy	Whole, live microglia were gated as previously described (Gosselin et al. Science 2017). Mouse brain nuclei were gated on DAPI+ singlets and were then gated on Olig2+, NeuN+, and PU1+ populations as previously described (Nott et al. Nature Protocols 2021). For experiments examining SALL1 expression, PU1+ nuclei were subdivided into SALL1 negative and SALL1 positive populations.

☒ Tick this box to confirm that a figure exemplifying the gating strategy is provided in the Supplementary Information.
